# Uncovering Backbone Conformation for Rigid DPP-Based Donor–Acceptor Conjugated Polymer Using Deuterium Labeling and Neutron Scattering

**DOI:** 10.1021/acs.macromol.4c01496

**Published:** 2024-10-21

**Authors:** Zhiqiang Cao, Zhaofan Li, Madison Mooney, Changwoo Do, Kunlun Hong, Simon Rondeau-Gagné, Wenjie Xia, Xiaodan Gu

**Affiliations:** † School of Polymer Science and Engineering, Center for Optoelectronic Materials and Devices, The University of Southern Mississippi, Hattiesburg, Mississippi 39406, United States; ‡ Department of Aerospace Engineering, Iowa State University, Ames, Iowa 50011, United States; § Department of Chemistry and Biochemistry, University of Windsor, Windsor, Ontario N9B3P4, Canada; ∥ Neutron Scattering Division, Oak Ridge National Laboratory, Oak Ridge, Tennessee 37831, United States; ⊥ Center for Nanophase Materials Sciences, Oak Ridge National Laboratory, Oak Ridge, Tennessee 37831, United States

## Abstract

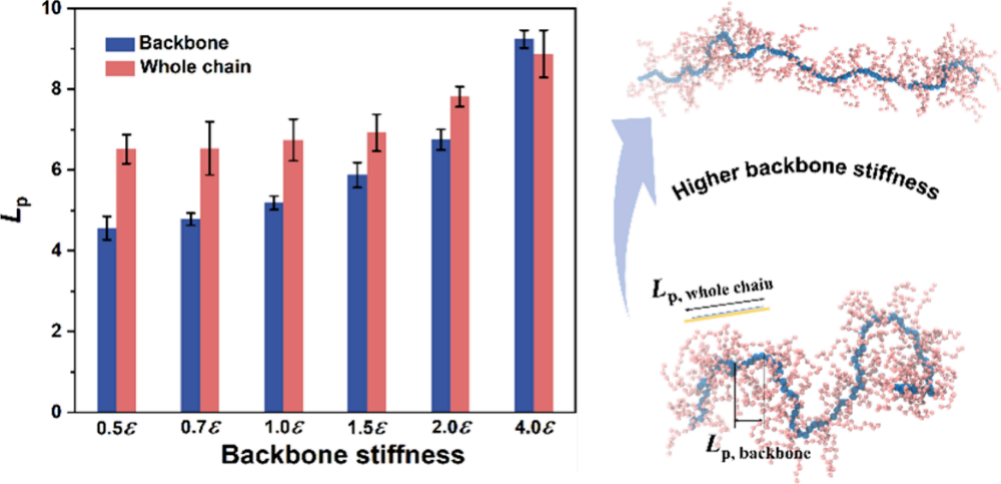

The conjugated polymer’s backbone conformation dictates the delocalization of electrons, ultimately affecting its optoelectronic properties. Most conjugated polymers can be viewed as semirigid rods with their backbone embedded among long alkyl side chains. Thus, it is challenging to experimentally quantify the conformation of a conjugated backbone. Here, we performed contrast variation neutron scattering on rigid conjugated donor–acceptor (D–A) diketopyrrolopyrrole (DPP) polymers with selectively deuterated side chains to measure the conjugated backbone conformation. We first synthesized DPP-based polymers with deuterated side chains, confirmed by NMR and FTIR. Using contrast variation neutron scattering, we found that the DPP-based conjugated polymers are much more rigid than poly(3-alkylthiophenes), with persistence length (*L*_p_) at 16–18 nm versus 2–3 nm. More importantly, in contrast to the relatively flexible poly(3-alkylthiophenes) whose backbone is more flexible than the whole polymer, we found that the backbone of DPP-based polymers has the same *L*_p_ value compared to the whole polymer chain. This indicates that side chain interference on backbone conformation is not present for the semirigid polymer, which is further confirmed by coarse-grained molecular dynamics (CG-MD) simulations. Our work provides a novel protocol to probe polymer’s backbone conformation and paradigm-shifting understanding of the backbone conformation of semirigid conjugated polymers.

## Introduction

Since adopted in 1987 by Reza Oboodi et al.,^1^ grafting flexible side groups to rigid conjugated backbones has been widely used not only to endow solution processability to semiconducting polymers but also to enable tunable mechanical properties^2^ and optoelectronic performances,^3^ facilitating a wide range of applications for optoelectronic devices.^4−10^ For the design of these materials, large bulky alkyl side chains are most commonly used to improve the solubility of the polymer in common organic solvents, hence enabling their processing through solution deposition techniques. In addition, these side chains are easily accessible and tunable synthetically, and they have well-known effects on various properties of the semiconducting polymer. Because of this design, conjugated polymers (CPs) are commonly viewed as semirigid polymers with highly heterogeneous structures and segmental dynamics. Recently, the high charge carrier mobilities of donor–acceptor (D–A) type semiconducting CPs have been accredited to the rigidity and planarity of the π-conjugated backbone that facilitates the delocalization of electrons along the polymer backbone.^11,12^ Thus, it is important to investigate the conformation of the conjugated backbone of D–A copolymers to further develop next-generation high-performance optoelectronic materials.

In the literature, reports of the rigidity of D–A conjugated polymers are frequently based on the use of scattering tools to probe the shape of whole polymeric chains rather than focus solely on the conjugated backbone.^13−15^ However, for semirigid polymers, the conformation of the backbone and whole polymer chain can be very different, especially for macromolecules with large amount of bulky side chains.^16^ For example, a recent work on traditional bottlebrush polymers (copolymers of methacrylate-terminated polydimethylsiloxane with methyl methacrylate) with flexible backbones showed that the backbone of the bottlebrush polymer can even fold into a cylindrical core.^17^ The backbone folding induced an increased bottlebrush diameter with decreasing side chain grafting density, driven by the incompatibility between side chains and the backbone polymer.^17^ In this case, the conformation of the entire polymer chain is not representative of the conformation of the backbone. Thus, the ability to decouple the backbone and side chain conformation could have important implications for designing responsive and functional bottlebrush materials including polymer networks with extreme stretchability^18^ and unusual dynamic mechanical properties.^19^ Recently, our group successfully measured the backbone conformation of poly(3-alkylthiophene) polymers. By deuterating the alkyl side chains, the scattering signal from the electronically functional conjugated backbone can be directly measured using contrast-variation small-angle neutron scattering (CV-SANS) in a good solvent. Our work indicated that the backbone is much more flexible than whole polymeric polymers by a factor of 2–3 times.^20^

In recent years, longer and branched side chains have been extensively studied to promote the solubility of D–A conjugated polymers.^21^ These D–A polymers are expected to be much more rigid than poly(3-alkylthiophenes), which has been believed to be one of the reasons why D–A polymers demonstrated superior electrical performance compared to poly(3-alkylthiophenes). Thus, many conclusions cannot be transferred directly from poly(3-alkylthiophenes) to D–A polymers with more complex and heterogeneous molecular structures.^22^ The dependence of persistence length (*L*_p_), the length where the orientation of the chain remains correlated, on side chain size is different for poly(3-alkylthiophenes) and D–A conjugated polymers. For example, poly(3-dodecylthiophene) (P3DDT) has a significantly lower *L*_p_ than poly(3-hexylthiophene) (P3HT) in solution, at 1 nm versus 3 nm, respectively.^23^ By contrast, the larger alkyl side chains of cyclopentadithiophene-*co*-pyridalthiadiazole polymers (PCPDTPT),^12^ diketopyrrolopyrrole (DPP)-based polymers,^14^ and quaterthiophene-*co*-(difluorinated) benzothiadiazole (PffBT4T) polymers^13^ all lead to a higher *L*_p_. However, no backbone *L*_p_ values have yet been measured for the D–A CPs. The lack of side chain deuterium-labeled D–A CPs and underutilized contrast variation neutron scattering have left fundamental gaps in our knowledge of the backbone conformation of D–A CPs.

Here, we successfully elucidated the backbone conformation of DPP-based CPs by combining neutron scattering and selective side chain deuterium labeling. For the first time, we successfully synthesized deuterated long and branched alkyl side chains specific for DPP-based polymers using Pd- and Pt-catalyzed H/D exchange reactions. Then the deuterated alkyl side chains were grafted to the DPP monomer, and DPP-based polymers were synthesized via Stille coupling reaction. Using contrast variation neutron scattering, we found that the backbone rigidity of the DPP polymer is equivalent to that of the whole polymer chain. MD simulations show that the difference between the backbone’s *L*_p_ and the whole polymer chain’s *L*_p_ decreases with increased backbone rigidity. Compared to relatively flexible poly(3-alkylthiophenes), the backbone of DPP-based conjugated polymers has a more anisotropic chain shape (e.g., *L*_p_ is much larger than the radius of the polymer); thus, the *L*_p_ of DPP CPs is similar to its backbone *L*_p_. Our work shows that integrated deuteration chemistry and neutron scattering protocols can probe polymer’s backbone conformation, and our findings provide a paradigm-shifting understanding of the backbone conformation of semirigid conjugated polymers.

## Experimental Section

### Materials

Reactants and solvents were purchased from Sigma-Aldrich. Commercial reactants were used without further purification unless stated otherwise.

### Synthesis of Side Chain Deuterated DPP-Based Polymers

Deuterium-labeled alkyl side chains were synthesized by a Pd- and Pt-catalyzed H/D exchange reaction.^24−26^ See the Supporting Information for synthetic details for side chain deuterated DPP-based polymers.

### Characterization

Nuclear magnetic resonance (NMR) spectra were obtained by using a Bruker Neo high-field NMR spectrometer operating at 500 MHz. Chemical shifts were recorded in ppm relative to CDCl_3_ at 7.26 ppm for ^1^H NMR and 77.17 ppm for ^13^C NMR. Fourier transform infrared (FTIR) spectroscopy was recorded in transmission mode by using a Bruker LUMOS II spectrometer. The polymer was coated to a KBr disk, and 128 scans were collected at 2 cm^–1^ resolution. A background spectrum of the KBr disk was also obtained using 128 scans at the same resolution as that for reference. The baseline was corrected by using the built-in instrument software package (OPUS). The number-average molecular weight (*M*_n_) and dispersity (*Đ*) were determined using gel permeation chromatography (GPC) relative to polystyrene standards at 160 °C in 1,2,4-trichlorobenzene (stabilized with 125 ppm of butylated hydroxytoluene). An Agilent PL-GPC 220 high-temperature GPC/SEC system equipped with four PLgel 10 μm MIXED-B columns was utilized for the analysis. Small-angle neutron scattering (SANS) was employed to investigate the conformation of single polymer chains. Dilute solutions of DPP polymer (5 mg mL^–1^) in a mixture of protonated solvents (*o*-DCB) and deuterated solvents (*o*-DCB-d4) were prepared. The percentage (v/v) of *o*-DCB-d4 varied from 0 to 100% to tune the SLD of solvent mixtures. SANS measurements were carried out at the extended Q-range small-angle neutron scattering diffractometer (EQ-SANS) at the Spallation Neutron Source (SNS), Oak Ridge National Lab (ORNL). The scattering wavevector ranged from 0.003 to 0.7 Å^–1^, using two different instrumentation configurations (4 m sample-to-detector distance with a wavelength band of λ_min_ = 12 Å and 2.5 m sample-to-detector distance with λ_min_ = 2.5 Å). A clean wavelength band of 3–4.3 Å wide is achieved by three bandwidth choppers on the EQ-SANS. The solution samples were housed in Hellma quartz cells with a path length of 2 mm path length. SANS measurements were conducted at 130 °C. Data reduction and correction for absolute intensity were performed, and the results were placed on an absolute scale by incorporating the differential cross section per unit volume of Porasil (cm^–1^). UV–vis–NIR spectroscopy was obtained by using a Cary 5000 UV–vis–NIR spectrophotometer. Solution absorption data were collected from polymer solutions in *o*-DCB solvent (0.1 mg mL^–1^) with temperatures spanning from 25 to 160 °C.

### Overview of CG-MD Simulation

We characterized the structural attributes of the polymer backbone and side chains utilizing a simplified bead–spring coarse-grained (CG) model featuring branched chain configurations, aiming to retain the crucial structural characteristics. Specifically, the polymer backbone, represented in blue, is accompanied by side chains, each comprising *N*_sc_ = 10 segments represented in pink, in alignment with the model setup established in our previous work (see Figure 4a for the model).^20^ To maintain generality throughout our study, all physical quantities are represented in reduced Lennard-Jones (LJ) units within our system. To replicate the good-solvent solution state, nonbonded pair interactions are implemented via a cut-and-shifted LJ potential:^27,28^
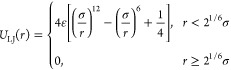
where σ and ε denote the units of length and energy, respectively. The potential energy associated with the bond stretching is described by a harmonic bonding potential *U*_bond_(*r*) = *K*(*r* – *r*_0_)^2^, where *K* = 2500ε/σ^2^ refers to the stiffness constant and *r*_0_ = 0.97σ is the equilibrium bond length, consistent with previously investigated branched polymers.^29,30^ Specifically, chain stiffness is regulated using a three-body angular potential defined as *U*_angle_(θ) = *k*_θ_(1 + cos(θ)), where angular stiffness constant *k*_θ_ is varied from 0.5ε to 4.0ε to adjust the stiffness of the backbone. For the side chains, *k*_θ_ is set to 0.2ε. To examine the impact of polymer length on persistence lengths, we tested different chain lengths ranging from *n* = 20 to 100 (Figure S5). Notably, *n* = 20 corresponds to DPP polymers observed in experiments, where the contour length is approximately twice the persistence length. Even for *n* = 100, where the contour length is ten times larger, the persistence lengths of the backbone and the whole polymer remain closely aligned. These findings demonstrate that polymer length does not significantly alter the persistence length relationship, addressing concerns about large persistence lengths relative to the contour length. Similar CG models have been extensively employed in the exploration of a diverse range of dynamic and structural properties inherent to CPs, in both their melt and solution phases, emphasizing the established utility of such models in the realm of CP research.^2,31−33^ Our previous work^20^ has provided comprehensive model details, rendering our current description supplementary. It is important to note that the force field parameters employed herein were utilized in a qualitative capacity to depict the rough molecular structure, as informed by experimental investigations of CPs.

## Results

### Synthesis of Deuterated DPP-Based Polymers

**Figure 1:**
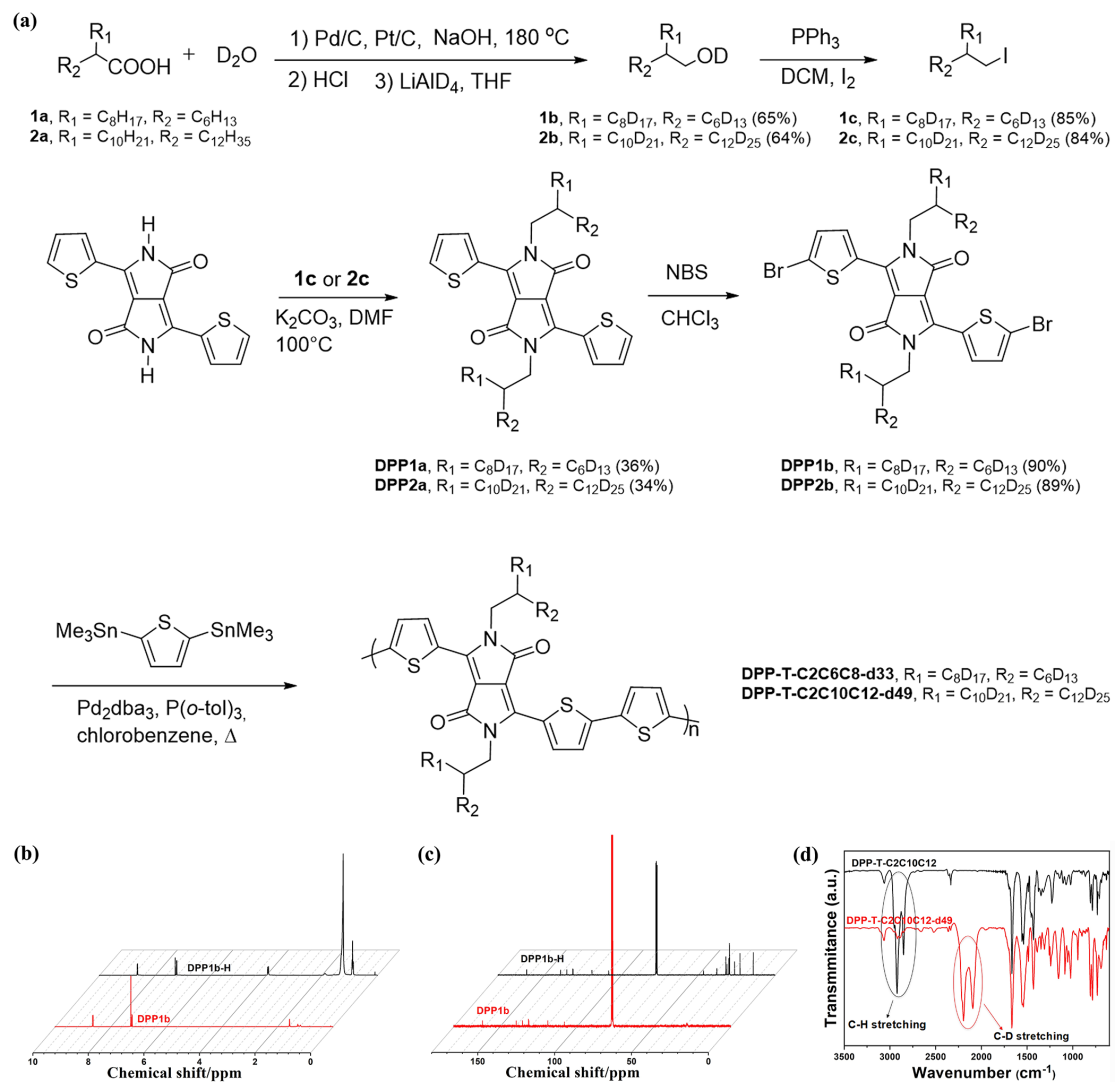
Synthesis of deuterated side chains and deuterated DPP polymers. (a) Synthetic route of deuterated alkyl side chains, DPP-T-C2C10C12 and DPP-T-C2C10C12-d49. (b) ^1^H and (c) ^13^C NMR spectra of monomer DPP2b and DPP2b-H (nondeuterated). (d) FTIR spectra of DPP-T-C2C10C12 and DPP-T-C2C10C12-d49.

We first synthesized DPP polymers with deuterated alkyl side chains using the synthetic route shown in Figure 1a. Pd- and Pt-catalyzed H/D exchange was adopted for the synthesis of deuterated alkyl side chains.^26^ The detailed synthetic steps can be found in the Supporting Information. To achieve a high deuteration ratio, 3 to 5 rounds of H/D exchange are required. Table 1 summarizes the molecular weight, dispersity, and deuteration ratio of the synthesized DPP-based polymers.

**Table 1:** Summary of the Materials Property of Synthesized DPP Polymers

polymer	*M*_n_ (kDa)	*Đ*	deuteration ratio (%)
DPP-T-C2C6C8	62.8	2.48	0
DPP-T-C2C6C8-d33	71.7	2.18	96
DPP-T-C2C10C12	60.6	2.44	0
DPP-T-C2C10C12-d49	73.0	2.88	98

^1^H NMR and ^13^C NMR spectroscopy further confirmed the successful alkylation of the deuterated side chains to the DPP core (Figures 1b and 1c). Compared with protonated DPP2b-H, no clear proton peaks ascribed to H of alkyl side chains were observed for DPP2b from ^1^H NMR spectra at 1–4 ppm (Figure 1b). Additionally, as shown in the ^13^C NMR spectra (Figure 1c), for DPP2b, no single peak for all side chain carbons (10–50 ppm) can be seen.^34^ Further NMR analysis on the residual proton signal intensity can determine the deuteration ratio.^35^ The deuteration ratio exceeds 98%, as indicated by the integrated area of the residual ^1^H peaks and fully protonated CH_2_ and CH_3_; see the Supporting Information for NMR integration.

Deuteration of DPP-T-C2C10C12-d49 polymer was confirmed by Fourier transform infrared (FTIR) spectra (Figure 1d), as evidenced by the absence of alkyl C–H vibration and the appearance of alkyl C–D vibration. The absorption bands of DPP-T-C2C10C12 polymer at 2950–2850 cm^–1^ are attributed to the asymmetric C–H stretching vibrations in methyl groups and methylene groups of the alkyl chain as well as the symmetric C–H stretching vibration in methylene groups of the alkyl chain.^36,37^ In contrast, the asymmetric C–D stretching vibrations in −CD_3_ and −CD_2_, along with the symmetric C–D stretching vibration in –CD_2_, shift to 2300–2000 cm^–1^.^37^ The disappearance of C–H stretching bands further confirms the near-complete deuteration for DPP-T-C2C10C12-d49.

### Single-Chain Conformation of DPP Polymer in Dichlorobenzene Solvent Using Contrast-Variation SANS

**Figure 2:**
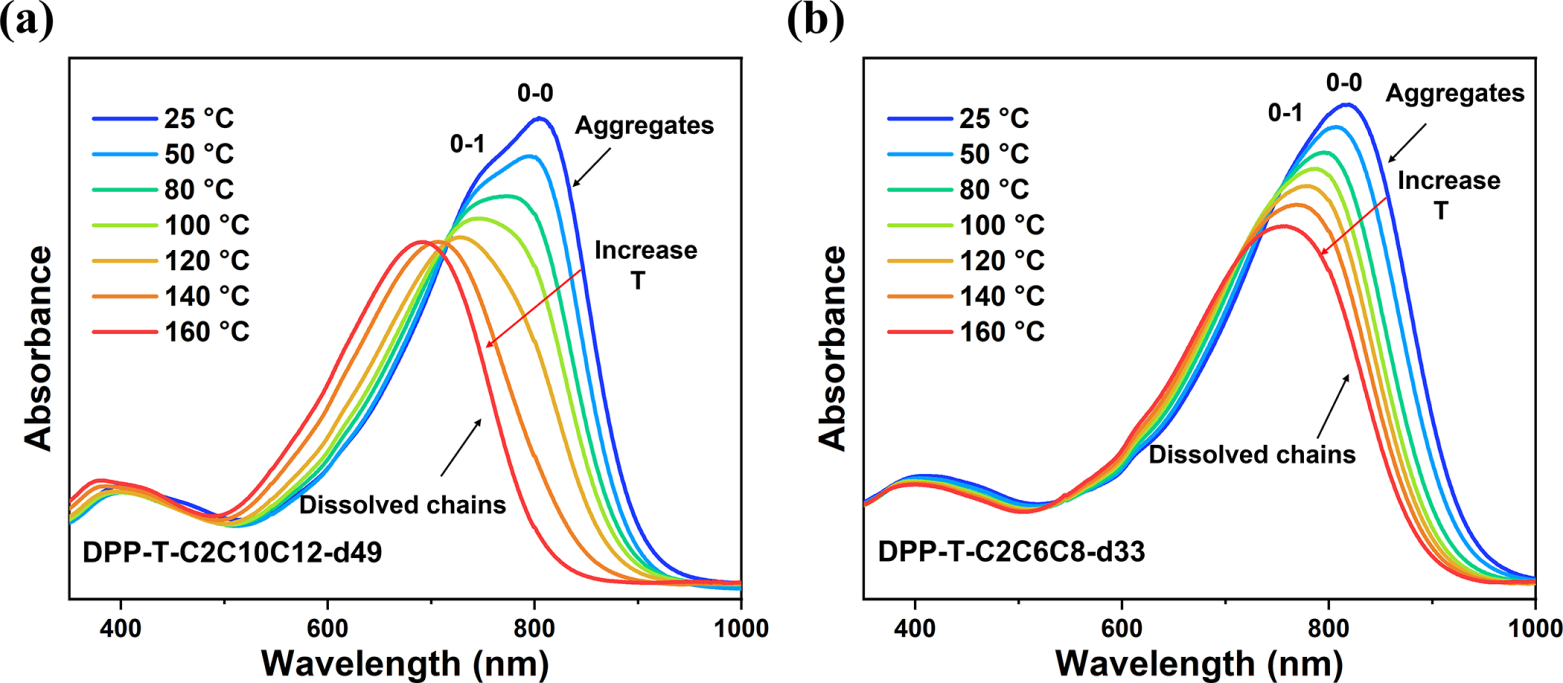
Temperature-dependent UV–vis of DPP-T-C2C10C12-d49 (a) and DPP-T-C2C6C8-d33 (b) solutions in *o*-DCB (0.1 mg mL^–1^).

After confirmation of the successful synthesis, we performed contrast-variation SANS on the deuterated polymers in a single-chain state. Our previous work shows that nondeuterated DPP-based polymers can form fully dissolved single chains in solutions at temperatures above 130 °C, using multimodal variable-temperature scattering and spectroscopy tools.^14^ Here, we also utilized temperature-dependent UV–vis measurements to monitor aggregation behaviors of side chain deuterated DPP polymers. We found that side chain deuterated DPP polymers exhibit similar aggregation behaviors compared with protonated DPP polymers.^14^ As shown in Figure 2a,b, both DPP-T-C2C10C12-d49 and DPP-T-C2C6C8-d33 polymers exhibited aggregation behaviors at room temperature. Upon heating, the primary absorption peak around 800 nm exhibits a gradual blue-shift, accompanied by a decreased intensity of 0–1 and 0–0 transition peaks, indicating that side-chain deuterated DPP polymer chains gradually disaggregate to single chains above 130 °C. Considering that the concentrations for UV–vis measurements and neutron scattering are different, we further performed variable-temperature ^1^H NMR on DPP-T-C2C10C12-d49 in deuterated dichlorobenzene at 5 mg mL^–1^. As shown in Figure S1, the broadening of the thiophene peaks is observed at low temperatures, and a clear resolution of the thiophene peaks is reached at 130 °C, which also indicates that DPP-T-C2C10C12-d49 can be fully dissolved at a concentration of 5 mg mL^–1^.^38^

**Figure 3:**
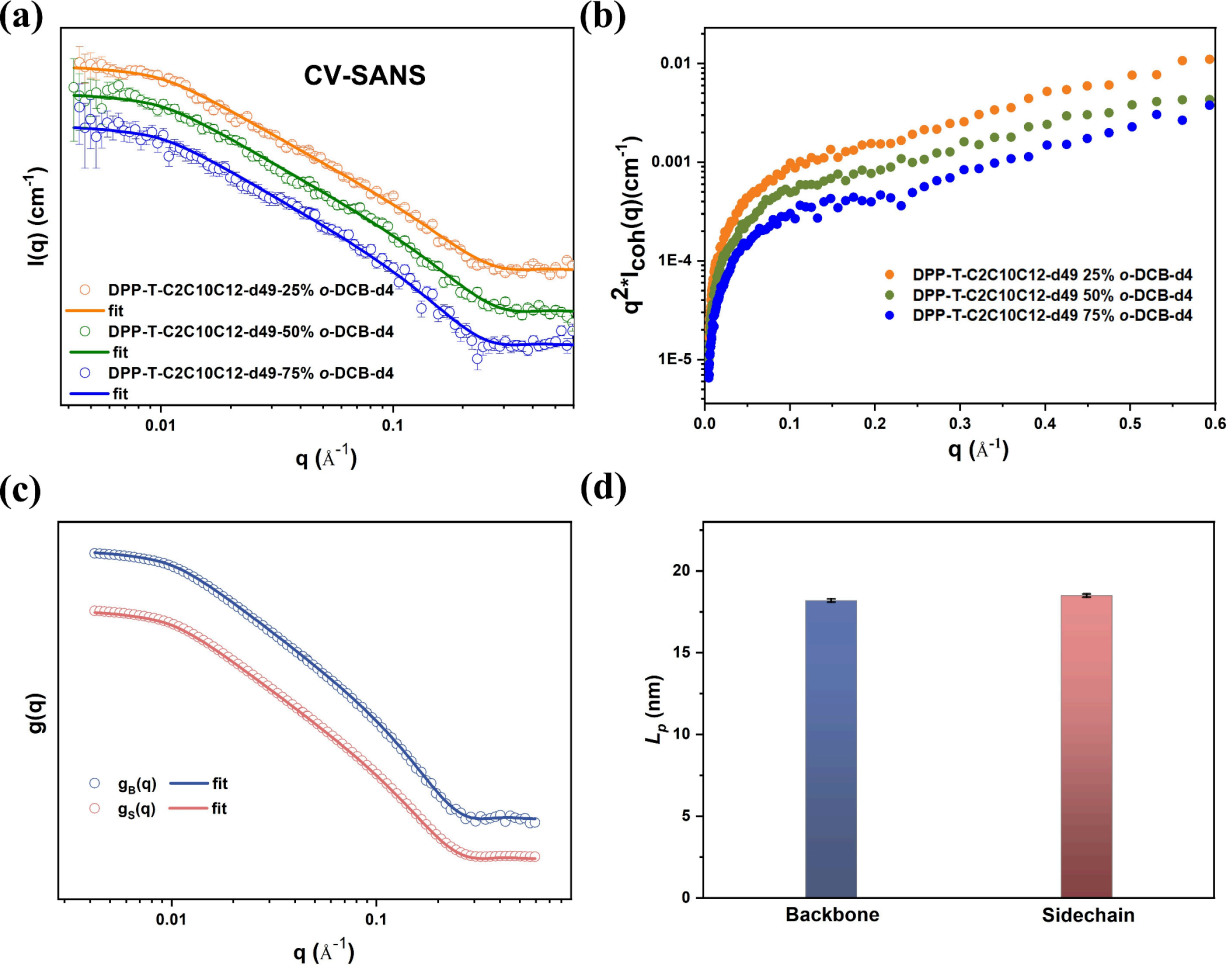
Contrast-variation scattering experiments for investigating backbone conformation. (a) Scattering profiles for DPP-T-C2C10C12-d49 from three different CV conditions at a concentration of 5 mg mL^–1^ at 130 °C. Solid lines are the best fit from the flexible cylinder model. (c) Kratky plots for DPP-T-C2C10C12-d49. (c) Form factors *g*_B_(*q*) and *g*_S_(*q*) obtained by solving eq 1. Solid lines represent the best fit to the flexible cylinder model. (d) *L*_p_ of backbone; side chain obtained from fitting to correlation functions of *g*_B_(*q*) and *g*_S_(*q*) of DPP-T-C2C10C12-d49 using the flexible cylinder model.

Contrast-variation SANS is a powerful technique to decipher the complex structure of complex materials, such as biological materials^39,40^ and polymer materials,^41^ especially when combined with deuterium labeling. The ideal case is to fully contrast match side chains of the polymer to get scattering signals from only the backbone. However, once deuterated, SLD for alkyl side chains increases to 7.57 × 10^–6^/Å^–2^ (a density of 1 g/cm^3^ for side chains was used for the calculation), which means the deuterated side chains cannot be fully matched in mixtures of *o*-DCB-d4 and *o*-DCB (SLD for deuterated DCB is 4.585 × 10^–6^/Å^–2^, which is even lower than deuterated side chains). Normally, when full contrast matching could not be achieved, CV-SANS measurements (at least three, and often five, CV conditions with widespread ratios of deuterated solvents are used) can also be utilized to separate backbone scattering from side chain scattering. We previously performed CV-SANS measurements on side-chain deuterated poly(3-alkylthiophenes).^20^ Here we adopt the same CV-SANS measurements at 130 °C for DPP-T-C2C10C12-d49 in mixtures of *o*-DCB and *o*-DCB-d4 solvents at different mixing ratios. Representative CV-SANS curves of DPP-T-C2C10C12-d49 under three CV conditions are shown in Figure 3a. For three CV experiments, the scattering profile of DPP-T-C2C10C12-d49 showed a Guinier region at low *q* range (0.004–0.008 Å^–1^). Kratky plots (Figure 3b) help to determine that DPP-T-C2C10C12-d49 polymers in different CV experiments are not flexible chains, as evidenced by the high-*q* upturn. In contrast, it should be a plateau for flexible polymer chains in this region. The power law analysis could provide more information. As shown in Figure S2, the scattering intensity decreases approximately by *q*^–1^ (0.01 Å^–1^ < *q* < 0.07 Å^–1^) which suggests that the polymer chains are indeed very rigid and close to rigid-rod behavior. The SANS curves of DPP polymers can be well fitted with a flexible cylinder model which is widely used for conjugated polymers,^14,15,42^ and the obtained Kuhn length is very high, with a value of 37–38 nm, which is longer than the polymer length. The fitting parameters are listed in Table 2. Interestingly, the obtained *L*_p_ values did not differ under different CV conditions.

**Table 2:** Parameters Obtained from Fits to SANS Data with the Flexible Cylinder Model; Contour Length (*L*_c_), Persistence Length (*L*_p_), and Radius (*R*) of DPP-Based Polymers (the Dispersity for Contour Length Is 1)

polymer/solvent	*L*_c_ (nm)	*L*_p_ (nm)	*R* (nm)
DPP-T-C2C10C12-d49-25% *o*-DCB-d4	33.8 ± 3.1	18.5 ± 2.0	1.1 ± 0.1
DPP-T-C2C10C12-d49-50% *o*-DCB-d4	33.8 ± 3.1	18.6 ± 2.2	1.0 ± 0.1
DPP-T-C2C10C12-d49-75% *o*-DCB-d4	33.8 ± 3.1	18.5 ± 2.0	1.2 ± 0.1

The DPP-T-C2C10C12-d49 polymer can have two respective scattering components from their backbone and side chains due to their large difference in the scattering length density (SLD, 2.17 × 10^–6^/Å^–2^ for backbone and 7.57 × 10^–6^/Å^–2^ for side chains; a density of 1 g/cm^3^ for backbone and side chains was used for the calculation). Thus, under varying CV conditions, the scattering contribution of the backbone (*g*_B_(*q*)), side chains (*g*_S_(*q*)), and the cross term (*g*_BS_(*q*)) between the backbone and side chains can differ significantly due to changes in the contrast between polymer and solvent.

Using the coherent differential cross sections (fitted data were used to avoid the noises of experimental data) for three CV conditions obtained from dilute solutions and solving eq 1,^20^ which describes the contribution the scattering contribution of backbone and side chains at different contrast variation conditions, we have got the form factor *g*_B_(*q*) for the backbone and *g*_S_(*q*) for side chains, as shown in Figure 3c.
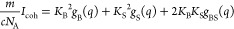
1where *I*_coh_ is the coherent scattering intensity, *m* represents the molar mass of monomers (g mol^–1^), *c* denotes the concentration of the polymer (g cm^–3^), *N*_A_ is Avogadro’s number (mol^–1^), and *K*^2^ (cm^2^) is the contrast factor (*K* = *v*(ρ – ρ_sol_), where ρ is the SLD of the different monomer parts of volume *v* and ρ_sol_ is that of the solvent) between the polymer and the solvent with the backbone denoted by symbol “B” and the side chains represented by symbol “S” (*K*_B_ = *v*_B_(ρ_B_ – ρ_sol_) and *K*_S_ = *v*_S_(ρ_S_ – ρ_sol_), *v*_B_ = 0.328 cm^3^ g^–1^ (378.44 cm^3^ mol^–1^), and *v*_S_ = 0.672 cm^3^ g^–1^ (1152.35 cm^3^ mol^–1^)). For varying CV conditions, the contrast factor values were calculated and are presented in Table 3. In this study, three CV conditions were established by adjusting the scattering length density (SLD) of the solvent using a mixture of deuterated and protonated solvents.

**Table 3:** Neutron Scattering Contrast Factors for DPP-T-C10C12-d49 in Different Solvents

contrast factor (10^–24^ cm^2^)	DPP-T-C10C12-d49 in 25% *o*-DCB-d4	DPP-T-C10C12-d49 in 50% *o*-DCB-d4	DPP-T-C10C12-d49 in 75% *o*-DCB-d4
*K*_B_^2^	0.059	0.182	0.372
*K*_S_^2^	9.832	7.617	5.685
2*K*_B_*K*_S_	–1.524	–2.354	–2.908

To determine the *L*_p_ of the backbone and side chains, we analyzed the form factors *g*_B_(*q*) and *g*_S_(*q*) by fitting them to a flexible cylinder model in SasView 5.0.5.^14,20^ The best fits for the flexible cylinder model are shown by solid lines in Figure 3c. The fitting parameters are listed in Table S1. The backbone of DPP-T-C2C10C12-d49 has a similar *L*_p_ as compared to the apparent *L*_p_ of the whole polymer, at 18.2 ± 0.1 versus 18.5 ± 0.1 nm (Figure 3d). Additionally, we previously conducted SANS on protonated DPP-T-C2C10C12 in *o*-DCB-d4 where the scattering originated from both the backbone and side chains. And the *L*_p_ obtained from fitting by the flexible cylinder model for the entire polymer chain was 18.0 ± 1.0 nm.^14^ Thus, the backbone *L*_p_ of DPP-T-C2C10C12-d49 is similar to that of the entire polymer chain, measuring 18.2 ± 0.1 nm compared with 18.0 ± 1.0 nm. The same phenomenon was also observed for DPP-T-C2C6C8-d33 (17.5 ± 0.1 versus 17.6 ± 0.1 nm); see Figure S4.

### CG-MD Simulations

**Figure 4:**
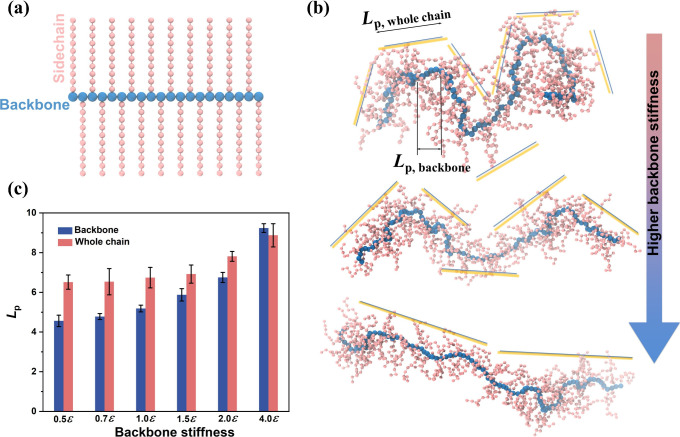
CG-MD simulations of chain conformation for semirigid polymer with long side chains. (a) Schematic of the CG model for the simulated CPs, showcasing a backbone comprising *N*_b_ = 100 segments (blue beads) and the side chains, each composed of *N*_sc_ = 10 segments (pink beads). (b) Representative snapshots of the simulated CP in a solution state, exhibiting varying degrees of backbone stiffness, where schematic yellow lines denote *L*_p,whole chain_. (c) Comparison of the persistence length (*L*_p_) for the backbone versus that for the overall polymer chain. All *L*_p_ values are reported in reduced LJ units.

Alongside our experimental investigations, we performed CG-MD simulations to deepen our understanding of the differences between the backbone conformation and the overall structure of the polymer chain. Based on the molecular structure of the DPP polymer, we adopted a generic bead–spring CG model that incorporates branched chain structures, composed of two primary constituents: a linear backbone chain (in blue), with each backbone bead being tethered to side chain beads (in pink), as shown in Figure 4a. The description of the CG model is provided in detail in the Experimental Section. One advantage of MD simulation is to characterize polymer chain behaviors with varying molecular parameters at a fundamental level that are typically challenging for experimental measurements. In our experimental par, the contour lengths of the DPP polymer is less than twice the persistence length. It is not entirely clear whether our conclusion, the persistence lengths of the backbone and whole polymer are the same for DPP polymers, also applies to longer polymer chains. DPP polymer chains are rigid, making it difficult to achieve a contour length significantly longer than the persistence length for several reasons. First, the synthesis of conjugated polymers using Stille or Suzuki coupling reactions is not a living polymerization process, making it more challenging to control the polymer’s molecular weight. Additionally, DPP polymers have a strong tendency to aggregate, which limits our ability to synthesize very long DPP polymers and conduct single-chain scattering experiments for high molecular weight polymers. When polymers aggregate, the scattering measures the aggregates rather than individual chains. Thus, we conducted MD simulations with varying numbers of repeat units, ranging from *n* = 20 to 100 (Figure S5). Notably, *n* = 20 corresponds to DPP polymers observed in experiments, where the contour length is approximately twice the persistence length. Even for *n* = 100, where the contour length is ten times larger, the persistence lengths of the backbone and the whole polymer remain closely aligned. These findings demonstrate that polymer length does not significantly alter the persistence length relationship, addressing concerns about large persistence lengths relative to the contour length. Of notable significance is the manipulation of chain stiffness, achieved through the implementation of a three-body angular potential described by *U*_angle_(θ) = *k*_θ_(1 + cos(θ)), wherein the angular stiffness constant, *k*_θ_, governs the rigidity of both the backbone and side chains. Specifically, we have systematically varied the *k*_θ_ parameter for the backbone across the range of 0.5ε to 4.0ε, while maintaining a fixed *k*_θ_ value of 0.2ε for the side chains, in order to capture different flexibility among polymer backbone and side chains. This deliberate variation in angular stiffness serves as a central component of our investigation, enabling a comprehensive exploration of the impact of backbone rigidity on chain conformation and rigidity.

In Figure 4b, we present three characteristic simulation snapshots of CPs with different backbone rigidity within a vacuum simulation cell to mimic their solution state. These snapshots corresponded to varying degrees of backbone rigidity: a highly flexible backbone (*k*_θ_ value of 0.5ε), a semirigid backbone (*k*_θ_ value of 1.0ε), and a highly rigid backbone (*k*_θ_ value of 4.0ε). Notably, in the case of a highly flexible backbone, the overall conformation of the polymer chain, incorporating both the backbone and side chains, exhibits significantly increased rigidity relative to that of the backbone alone, signifying a substantial disparity in *L*_p_. As the backbone stiffness increases, a notable extension in the chain’s conformation becomes apparent, as exemplified by the middle illustration in Figure 4b. In this scenario, characterized by a semirigid backbone, the difference between the overall contour of the polymer chain and the backbone alone is comparatively minor. Lastly, for the most rigid polymer model, the chain extends and adopts a nearly straight, rod-like configuration, resulting in a striking similarity between the overall conformation and the backbone-only representation, both displaying an extended and linear structure.

For a more comprehensive quantitative assessment of the chain conformation, we followed the same approach to calculate the *L*_p_ for both the backbone and the overall chain configuration, accounting for the presence of side chains. Specifically, to discern the impact of side chains on *L*_p_, we employed the center of geometry (COG) assumption, treating the center of geometry of a pair of side chains that connect with two adjacent backbone segments as a pseudochain, further contributing to the determination of an effective pseudochain for the overall CP contour.^20^ To ensure statistical reliability, we carried out eight independent simulations with varied initial configurations to compute the average value of *L*_p_, with the accompanying error bars representing standard deviations. In Figure 4c, we presented the results of *L*_p_ estimates for the backbone and the overall CP contour, while systematically varying the backbone stiffness parameter, *k*_θ_. Notably, in the case of the most flexible polymer backbone, our simulations reveal that the *L*_p_ of the backbone alone (e.g., 4.5 unit) is substantially smaller than that of the whole chain (6.5 unit), while the disparity between backbone rigidity and the overall polymer chain rigidity diminishes as backbone stiffness increases. Remarkably, in the most rigid polymer model, there is virtually no difference in *L*_p_ (e.g., both case showed 8.9 unit), aligning qualitatively with our experimental observations, where a significant *L*_p_ difference between the backbone and the entire chain is observed for the relatively flexible poly(3-decylthiophene) (P3DT),^20^ while this distinction is negligible for the comparatively rigid DPP-based CPs.

### Rigidity of the Polymeric Backbone for Semirigid Polymers

In this work, we discussed the measurement of the backbone conformation of semirigid conjugated polymers. In our work, we can view the conjugated polymer consisting of highly heterogeneous backbone segments and side chain segments. It is like another class of polymer, bottle brush materials, that has been studied extensively. Molecular bottlebrushes are realized to be able to integrate multiple properties into a single material, particularly for bottlebrush macromolecules featuring side chains and backbones made up of chemically distinct monomers. For example, one molecule, multiple strands graft polymers can be soft, firm, strong, or damping for a range of biomedical applications, including reconstructive surgery and wearable electronics.^43^ These multiple functions are closely related to the structural and dynamic heterogeneities of such bottlebrush macromolecules. So far, it is well-known that side chains can be effective diluents and stiffeners of backbones.^16^ However, it remains less understood in terms of the relationship between the whole bottlebrush conformation and that of the side chains and backbones.

To decouple backbone and side-chain conformations in conjugated polymers, we employed an approach that combines contrast-variation neutron scattering and deuterium labeling. Additionally, we conducted CG-MD simulations to confirm the distinctions between the backbone conformation and the overall conformation of the polymer chain. Our group’s series of works on conjugated polymers indicate that the inherent conformation of the electronically active conjugated backbone can be different from (or the same as) the conformation of the whole chain (depending on the relative size of the side chains and the persistence length of the backbone). The following conclusions stand out:

(i) When the persistence length of the backbone is comparable to the size of the side chains, like poly(3-alkylthiophenes) with a *L*_p_ of approximately 1–3 nm, our previous work indicated that the backbone is much more flexible than whole bottlebrush-like polymers.^20^
(ii) However, for semirigid conjugated polymers (e.g., DPP polymer in this case), its *L*_p_ is much larger than the size of the polymer side chains; thus, the effective *L*_p_ of DPP-based whole conjugated polymer chain is close to that of the backbone. In extreme cases, when the *L*_p_ is close to infinity (or perfectly rigid rod), one can imagine that there should be no difference between the overall chain and the backbone of the chain.

The classification of chain conformation presented here is based on conjugated polymers, which we believe can be extended to describe properties of other polymers with grafted side chains in solutions.

## Conclusions

In this work, we extended our previously established contrast-variation small-angle neutron scattering experiments to investigate the backbone conformation of much more rigid side-chain deuterated DPP-based polymers. Unlike the relatively flexible poly(3-alkylthiophenes), experimental results show that the backbone of DPP-based polymers has the same *L*_p_ compared to the whole polymer chain. MD simulations showed that the difference between backbone rigidity and whole polymer chain rigidity decreases with increasing backbone rigidity. The backbone has a *L*_p_ much larger than the side chain size; thus, the effective *L*_p_ of a DPP-based whole conjugated polymer chain is equal to that of the backbone. We believe that this conclusion can be extended to describe the properties of other polymers with grafted side chains in solutions. We anticipate that our discovery may catalyze the development of novel functional materials by leveraging control over the backbone and whole chain conformation.

## Supporting Information Available

NMR and MS spectra for all compounds and additional experimental results (PDF)
