# Supplementary material for: Uncovering Backbone Conformation for Rigid DPP-Based Donor–Acceptor Conjugated Polymer Using Deuterium Labeling and Neutron Scattering
Source: Macromolecules. 2024 Oct 21;57(21):10379–88. doi: 10.1021/acs.macromol.4c01496 (PMC11562799; doi:10.1021/acs.macromol.4c01496)
Supplement: Supplementary file 1 — ma4c01496_si_001.pdf [file ma4c01496_si_001.pdf]

Uncovering Backbone Conformation for Rigid DPP-Based Donor-Acceptor Conjugated Polymer Using Deuterium Labeling and Neutron Scattering

Zhiqiang Cao,[a] Zhaofan Li,[b] Madison Mooney,[c] Changwoo Do,[d] Kunlun Hong,[e] Simon Rondeau-Gagné,[c] Wenjie Xia,[b] Xiaodan Gu*[a]

a. School of Polymer Science and Engineering, Center for Optoelectronic Materials and Devices, The University of Southern Mississippi, Hattiesburg, MS 39406, USA. E-mail: xiaodan.gu@usm.edu

b. Department of Civil and Environmental Engineering, North Dakota State University, Fargo, ND 58108, USA.

c. Department of Chemistry and Biochemistry, University of Windsor, Windsor, Ontario, N9B3P4, Canada

d. Neutron Scattering Division, Oak Ridge National Laboratory, Oak Ridge, TN 37831, USA

e. Center for Nanophase Materials Sciences, Oak Ridge National Laboratory, Oak Ridge, TN 37831, USA.

Experimental Procedure

The atom% deuterium incorporation was determined by both MS and ^{1}H NMR spectrum. The method to determine atom% deuterium incorporation using MS is shown below. The incorporation of deuterium into each substrate was verified by MS, observing a shift in the isotope distribution. Using **DPP2a** as example: Isotope distribution (MS): 50.2%, **DPP2a**-d98, 16.3%, **DPP2a**-d97, 10.8%, **DPP2a**-d96, 7.2%, **DPP2a**-d95, 4.8%, **DPP2a**-d94, 3.3%, **DPP2a**-d93, 2.7%, **DPP2a**-d91, 1.8%, **DPP2a**-d90, 1.6%, **DPP2a**-d89, 1.2%, **DPP2a**-d88. Deuterium ratio = [(%d98x 98) + (%d97x 97) + (%d96x 96) + (%d95x 95) + (%d94x 94) + (%d93x 93) + (%d92x 92) + (%d91x 91) + (%d90x 90) + (%d89x 89)]/98 = 98%

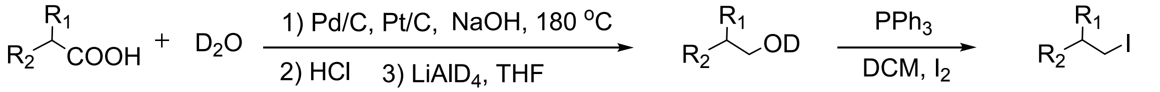

1a, $R_1 = C_8H_{17}$, $R_2 = C_6H_{13}$
2a, $R_1 = C_{10}H_{21}$, $R_2 = C_{12}H_{35}$

1b, $R_1 = C_8D_{17}$, $R_2 = C_6D_{13}$ (65%)
2b, $R_1 = C_{10}D_{21}$, $R_2 = C_{12}D_{25}$ (64%)

1c, $R_1 = C_8D_{17}$, $R_2 = C_6D_{13}$ (85%)
2c, $R_1 = C_{10}D_{21}$, $R_2 = C_{12}D_{25}$ (84%)

Scheme S1. Synthetic route of deuterated alkyl sidechains **1c** and **2c**.

General procedure for synthesis of 1b/2b: A 1-L stainless steel Parr reactor (series 4520) was charged with a solution of NaOH diluted with D_2O, followed by carboxylic acid compound **1a** or **2a**, and a combination of 10 wt% Pt/C and 10 wt% Pd/C catalysts. After assembly, the reactor was purged with nitrogen gas while stirring for 10mins, then purged with hydrogen gas for 1 min to activate the catalysts to reach a pressure of 10 pound-force per square inch (PSIG). The slurry was stirred (120 RPM) at 180 °C for 4 days, during which the pressure remained at 130 PSIG. After cooling, the reaction was quenched with hydrochloric acid solution (37 %), and dichloromethane was added to dissolve the alkyl carboxylic acids. The Pt and Pd catalysts were separated by extraction filtration. The liquid product was extracted with dichloromethane (3 x 200 mL), then the organic phase was dried over Na_2SO_4. After rotary evaporation, the product was subject to three additional cycles of Pd- and

Pt-catalyzed H/D exchange reaction to increase the deuteration level. Upon completion of these cycles, a 1-L round bottom flask, equipped with a magnetic pellet and fitted with a 200 mL constant pressure separator funnel carrying a take-off adapter, was flame-dried and cooled to room temperature, under inert nitrogen atmosphere. $LiAlD_4$ was added to the flask followed by anhydrous tetrahydrofuran. The solution was stirred in an ice bath for 1 hour. Deuterated carboxylic acid was dissolved in anhydrous tetrahydrofuran and added to constant pressure separator funnel then drip slowly into the ice bath cooled container. The reactor's temperature was then warmed to room temperature, and the solution was stirred overnight. The reaction was quenched with water and 10 % hydrochloric acid solution. The product was extracted with dichloromethane (3 x 500 mL) and the organic layer was dried using Na_2SO_4. After removal of solvents by rotary evaporation, the product was further purified by distilling under vacuum.

Compound 1b. NaOH (19.01 g, 47.5 mmol), D_2O (600.12 g), **1b** (40.00 g, 10.8 mmol), 10 wt% Pt/C (4.05 g), 10 wt% Pd/C (4.01 g), $LiAlD_4$ (25.25 g, 60.1 mmol), THF (600 mL). Yield of **1b**: 64 %. ^{1}H NMR (500 MHz, $CDCl_3$) δ 1.25 – 1.20 (m, J = 14.9, 8.0 Hz, residual H in methylene groups), 0.81 (d, *J* = 7.9 Hz, residual H in methyl groups). ^{13}C NMR (500 MHz, $CDCl_3$) δ 65.2, 65.1, 64.9, 64.7, 64.6, 39.8, 39.7, 39.6, 39.5, 39.4, 31.2, 31.1, 30.9, 30.8, 30.6, 30.5, 30.4, 30.2, 30.1, 29.9, 29.8, 29.7, 29.5, 29.3, 29.2, 28.9, 28.9, 28.8, 28.7, 28.6, 28.4, 28.3, 28.1, 28.0, 27.9, 26.3, 26.1, 26.1, 25.9, 25.4, 22.1, 22.0, 21.9, 21.9, 21.8, 21.7, 21.6, 21.6, 21.5, 21.3, 21.2, 13.8, 13.6, 13.5, 13.3, 13.2, 13.0, 12.9, 12.7, 12.6. Characterization of the compound by mass spectrometry was not possible due to difficulty in ionization.

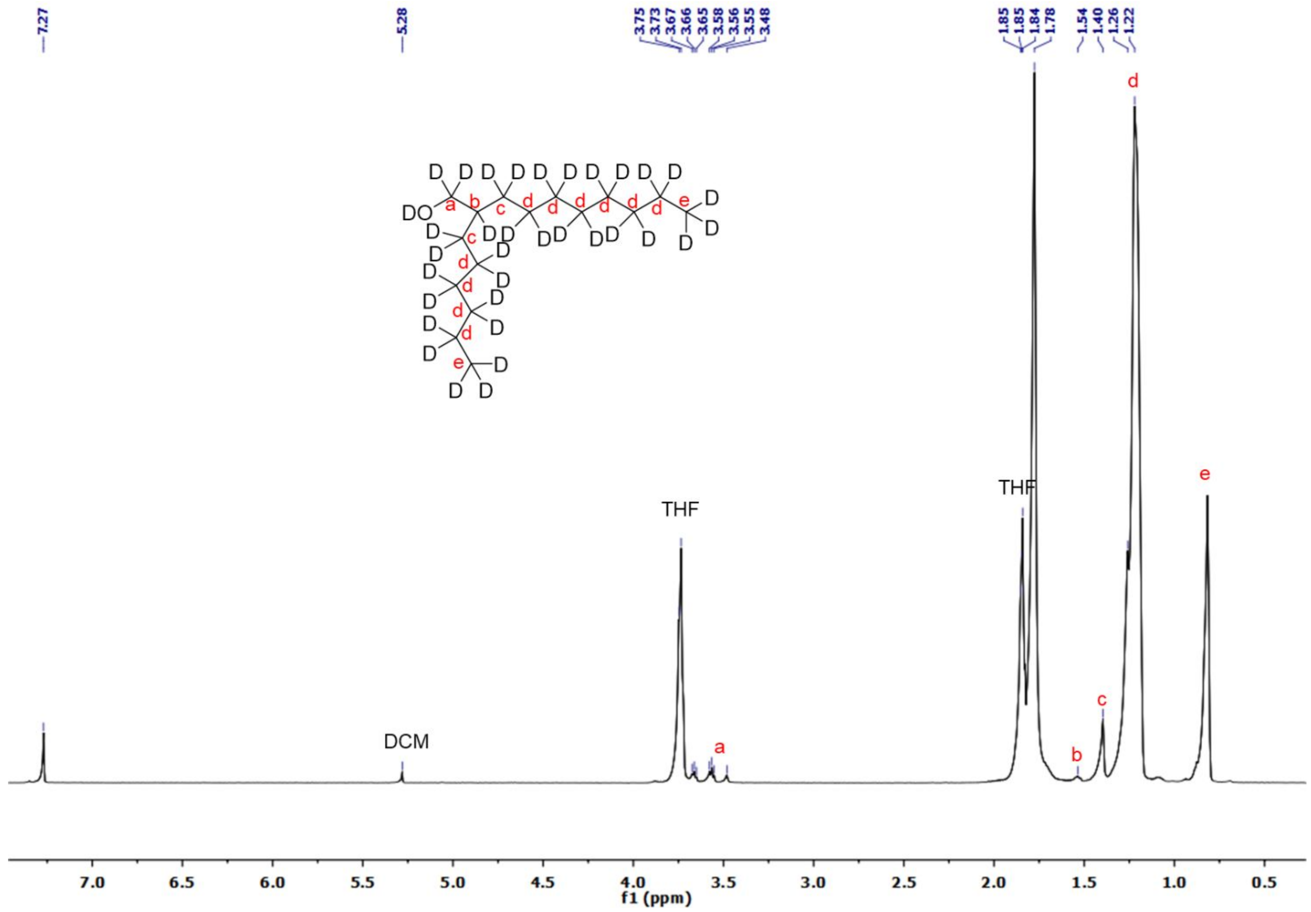

^{1}H NMR spectra of Compound **1b**

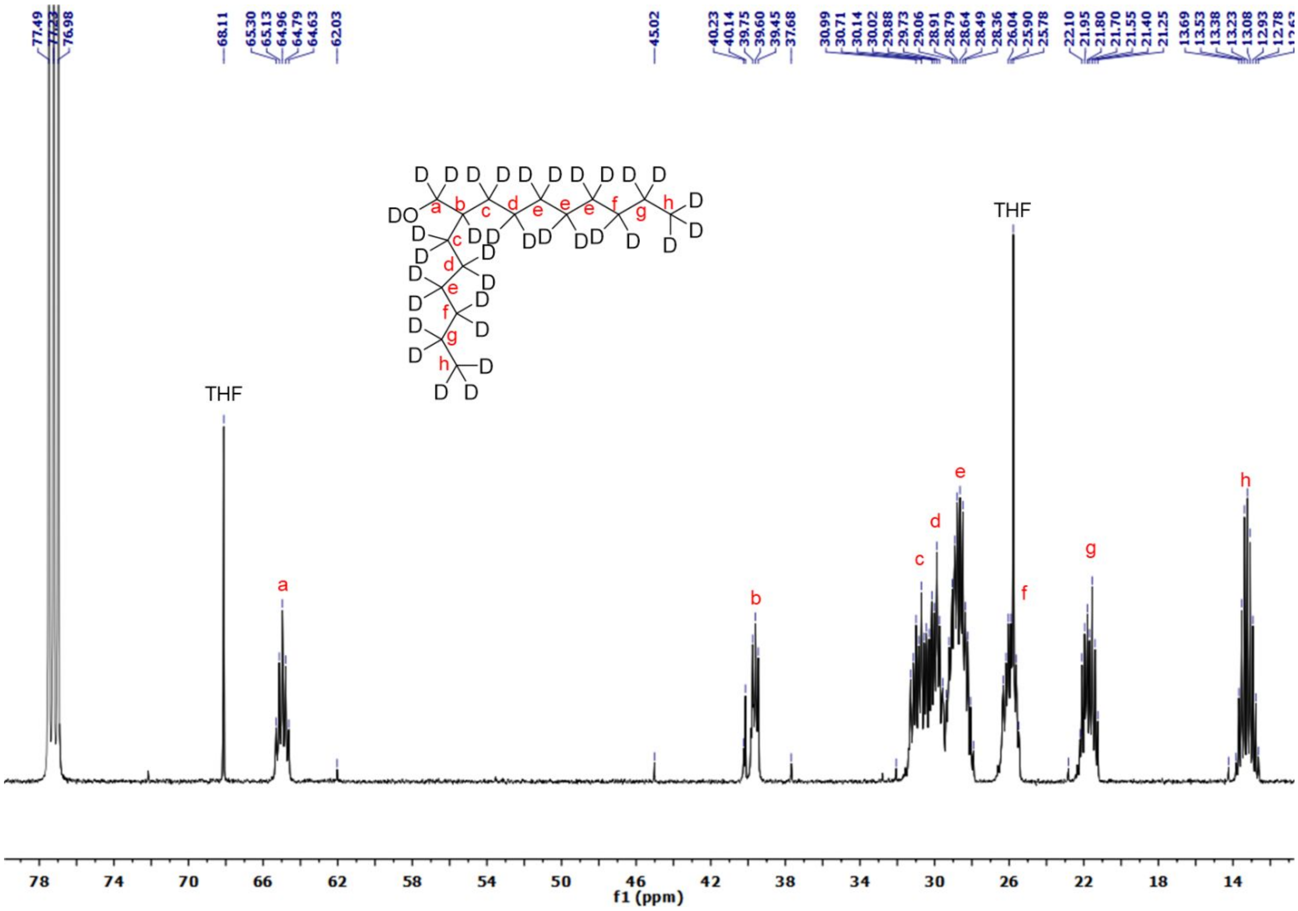

^{13}C NMR spectra of Compound **1b**

Compound 2b. NaOH (27.20 g, 67.9 mmol), D_2O (601.23 g), **2a** (40.04 g, 15.6 mmol), 10 wt% Pt/C (4.10 g), 10 wt% Pd/C (4.08 g,), $LiAlD_4$ (31.8 g, 75.8 mmol), THF (600 mL). Yield of **2b**: 65 %. ^{1}H NMR (500 MHz, $CDCl_3$) δ 1.26 (d, J = 5.6 Hz, residual H in methylene groups), 0.88 (t, J = 6.9 Hz, residual H in methyl groups). ^{13}C NMR (500 MHz, $CDCl_3$) δ 65.1, 64.9, 64.8, 39.7, 39.6, 39.4, 31.0, 30.9, 30.7, 30.6, 30.4, 30.1, 29.9, 29.9, 29.7, 29.5, 29.1, 28.9, 28.8, 28.7, 28.6, 28.5, 28.5, 28.4, 28.4, 28.2, 28.2, 28.1, 27.9, 26.1, 25.9, 25.7, 25.6, 25.5, 21.8, 21.7, 21.5, 21.4, 21.2, 13.5, 13.4, 13.2, 13.1, 12.9, 12.8. Characterization of the compound by mass spectrometry was not possible due to difficulty in ionization.

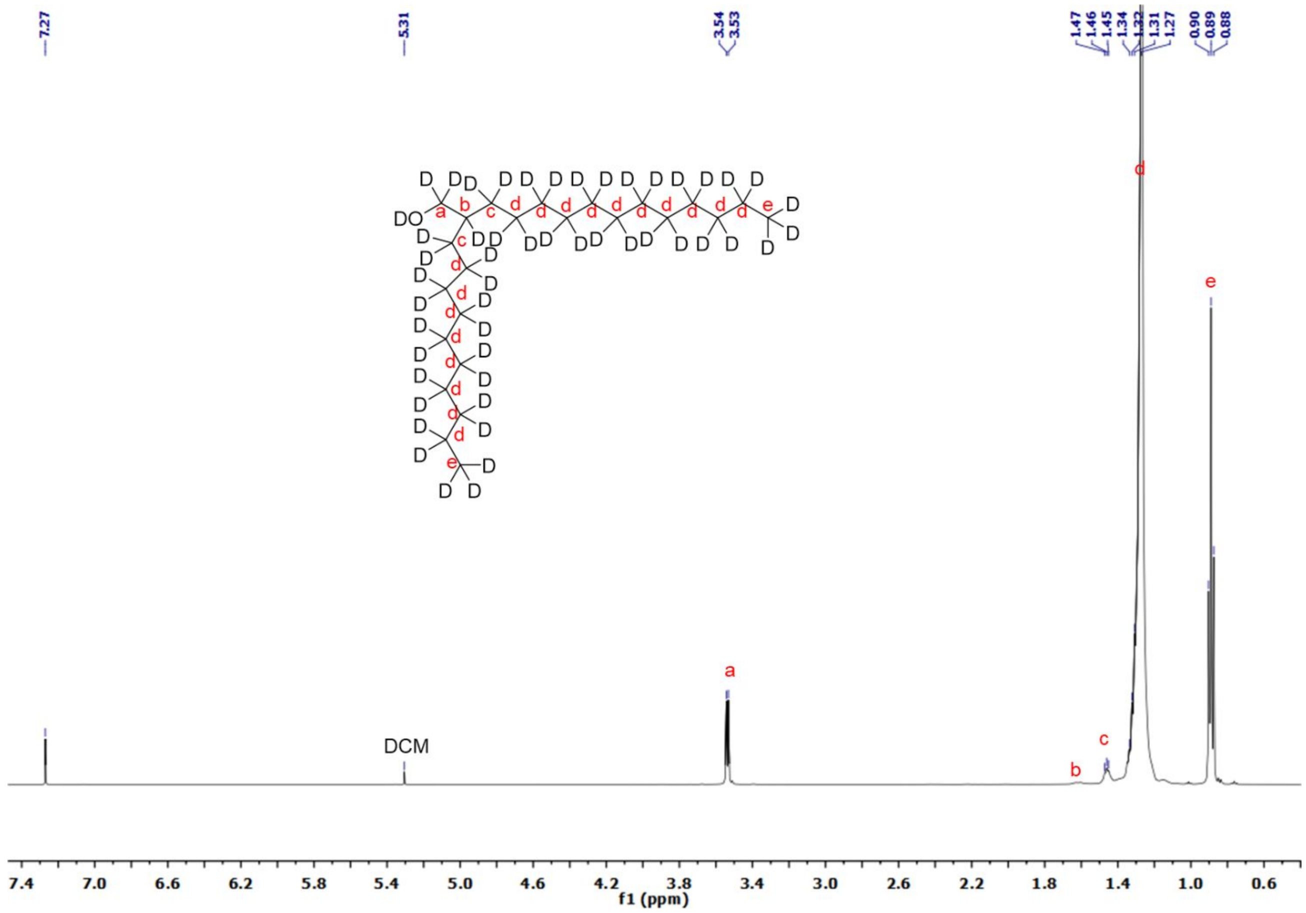

^{1}H NMR spectra of Compound **2b**

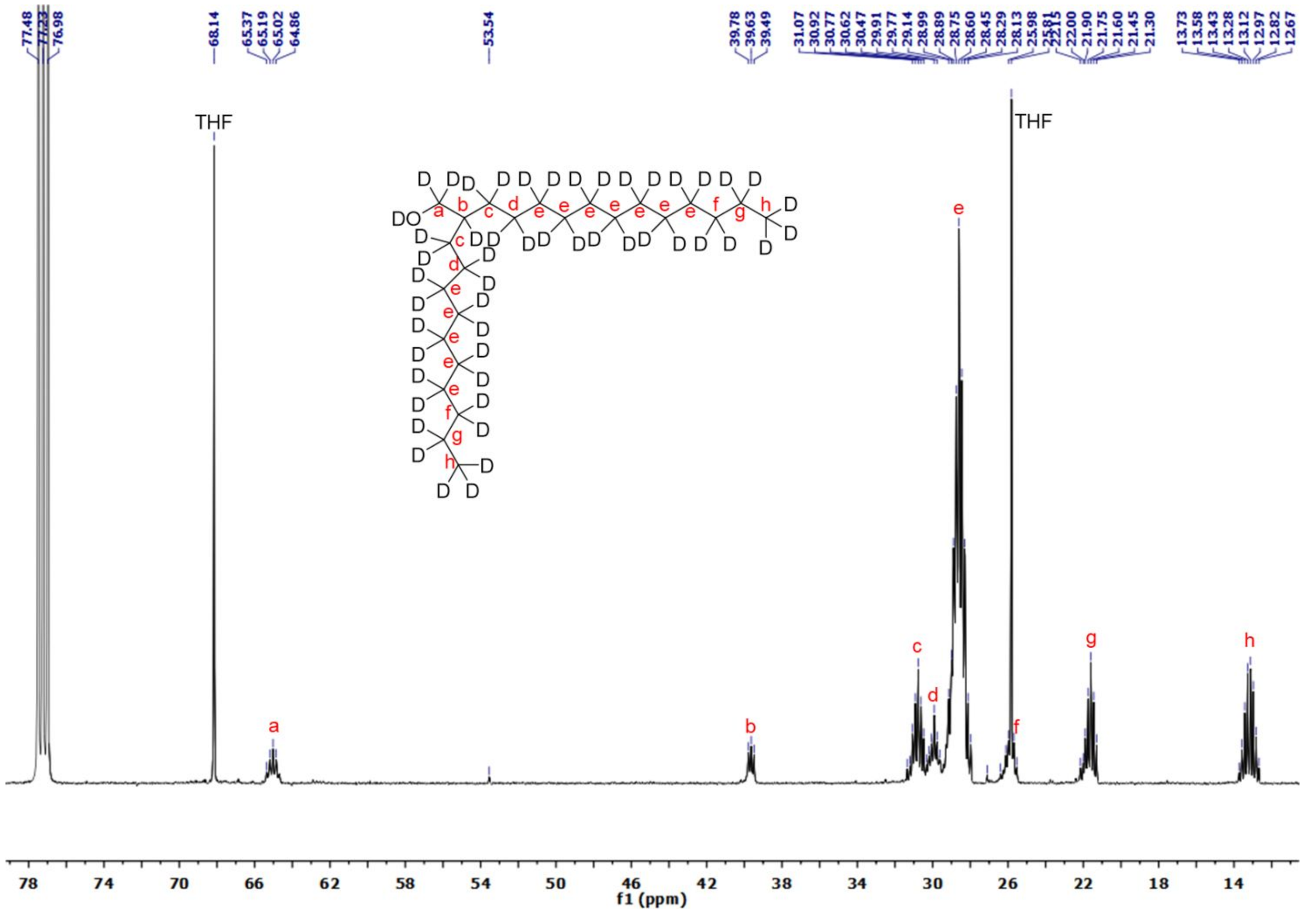

^{13}C NMR spectra of Compound **2b**

General procedure for synthesis of 1c/2c. A round-bottom flask equipped with a magnetic stir bar was purged, flame dried, and charged with the selected alcohol and anhydrous dichloromethane. The solution was stirred at room temperature under inert atmosphere until the compound was fully dissolved, then triphenyl phosphine and iodine were added. The reaction mixture was stirred under inert atmosphere for 15 hours at room temperature. Upon completion, the reaction was diluted in dichloromethane and extracted with H_2O. The organic layer was then washed with brine and dried using Na_2SO_4. The solvent was removed under reduced pressure and the crude mixture was purified by flash chromatography on silica gel using hexanes as eluent to afford the corresponding compound.

Compound 1c. 1b (10.00 g, 25 mmol), DCM (40 mL), PPh_3 (10.8 g, 41 mmol), I_2 (8.8 g, 35 mmol).

Yield of **1d**: 84 %. ^{1}H NMR (500 MHz, $CDCl_3$) δ 1.23 – 1.08 (m, J = 14.9, 8.0 Hz, residual H in methylene groups), 0.92 – 0.83 (m, residual H in methyl groups). ^{13}C NMR (500 MHz, $CDCl_3$) δ 37.9, 37.8, 37.7, 33.9, 33.7, 33.6, 33.5, 33.3, 33.2, 30.9, 30.9, 30.6, 28.9, 28.8, 28.7, 28.5, 28.4, 28.2, 28.1, 25.9, 25.8, 25.6, 25.5, 25.3, 25.2, 22.0, 21.9, 21.8, 21.5, 21.3, 16.5, 16.3, 16.2, 13.7, 13.5, 13.4, 13.2, 13.1, 12.9, 12.8. Characterization of the compound by mass spectrometry was not possible due to difficulty in ionization.

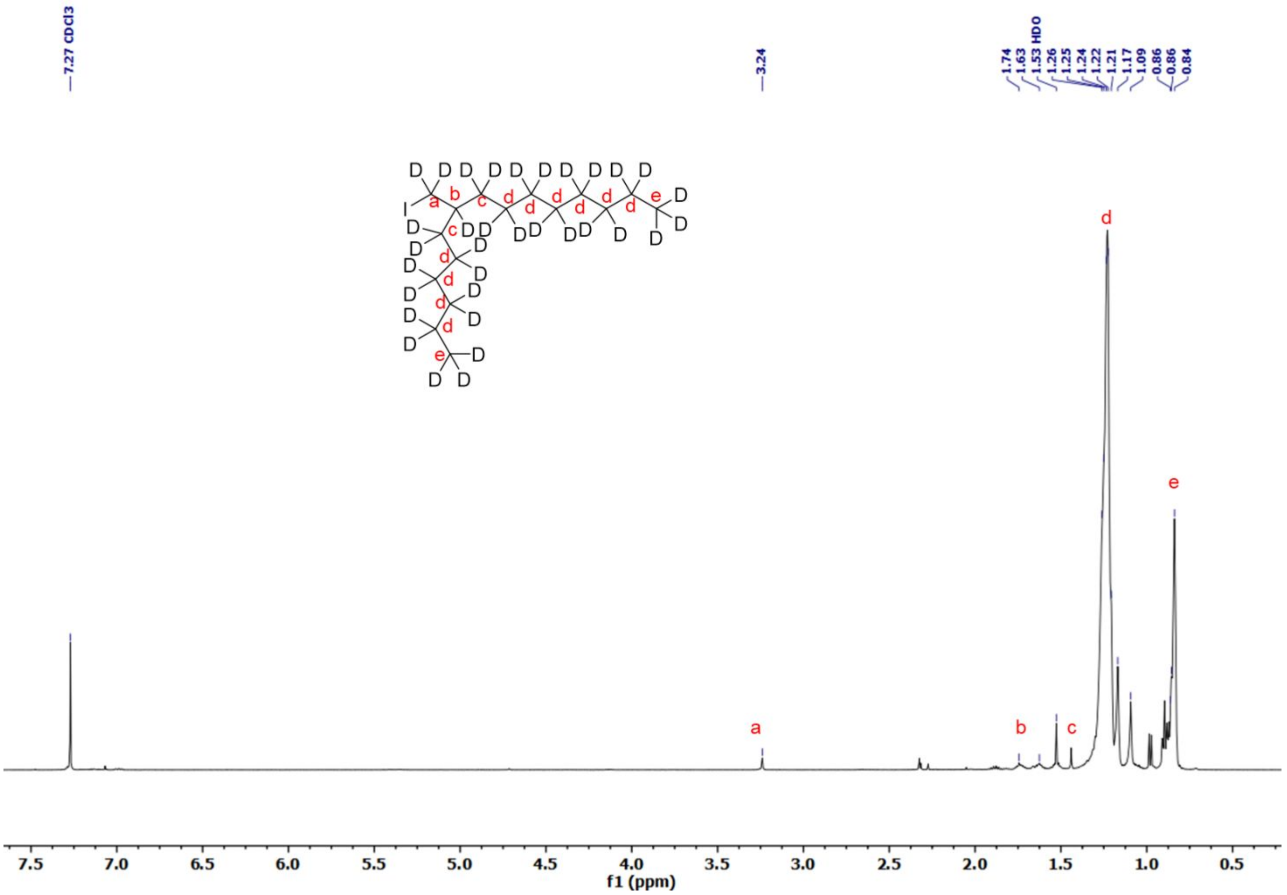

^{1}H NMR spectra of Compound **1c**

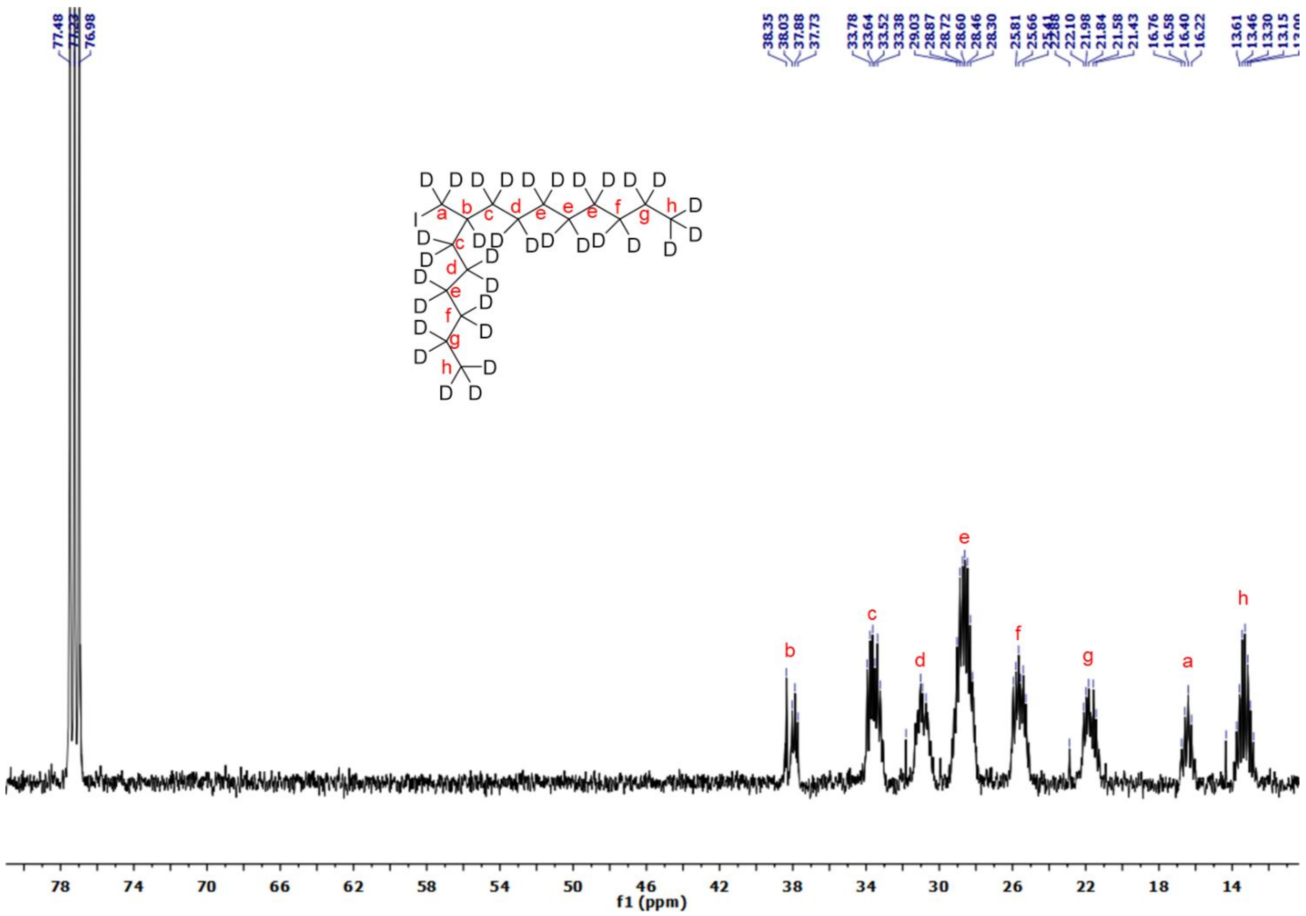

^{13}C NMR spectra of Compound **1c**

Compound 2c. 2b (10.00 g, 36.6 mmol), DCM (60 mL), PPh_3 (15.7 g, 59.8 mmol), I_2 (12.8 g, 51.1 mmol). Yield of **1d**: 85 %. ^{1}H NMR (500 MHz, $CDCl_3$) δ 1.31 – 1.20 (m, residual H in methylene groups), 0.88 (m, residual H in methyl groups). ^{13}C NMR (500 MHz, $CDCl_3$) δ 38.0, 37.9, 37.7, 33.6, 33.5, 33.4, 33.2, 31.2, 31.1, 30.9, 30.8, 30.6, 30.5, 29.2, 29.0, 28.9, 28.8, 28.7, 28.6, 28.6, 28.5, 28.4, 28.3, 28.3, 28.1, 27.9, 25.7, 25.6, 25.4, 25.3, 22.1, 21.9, 21.9, 21.7, 21.6, 21.4, 21.3, 16.3, 16.1, 15.9, 13.6, 13.4, 13.3, 13.1, 12.9, 12.8, 12.7. Characterization of the compound by mass spectrometry was not possible due to difficulty in ionization.

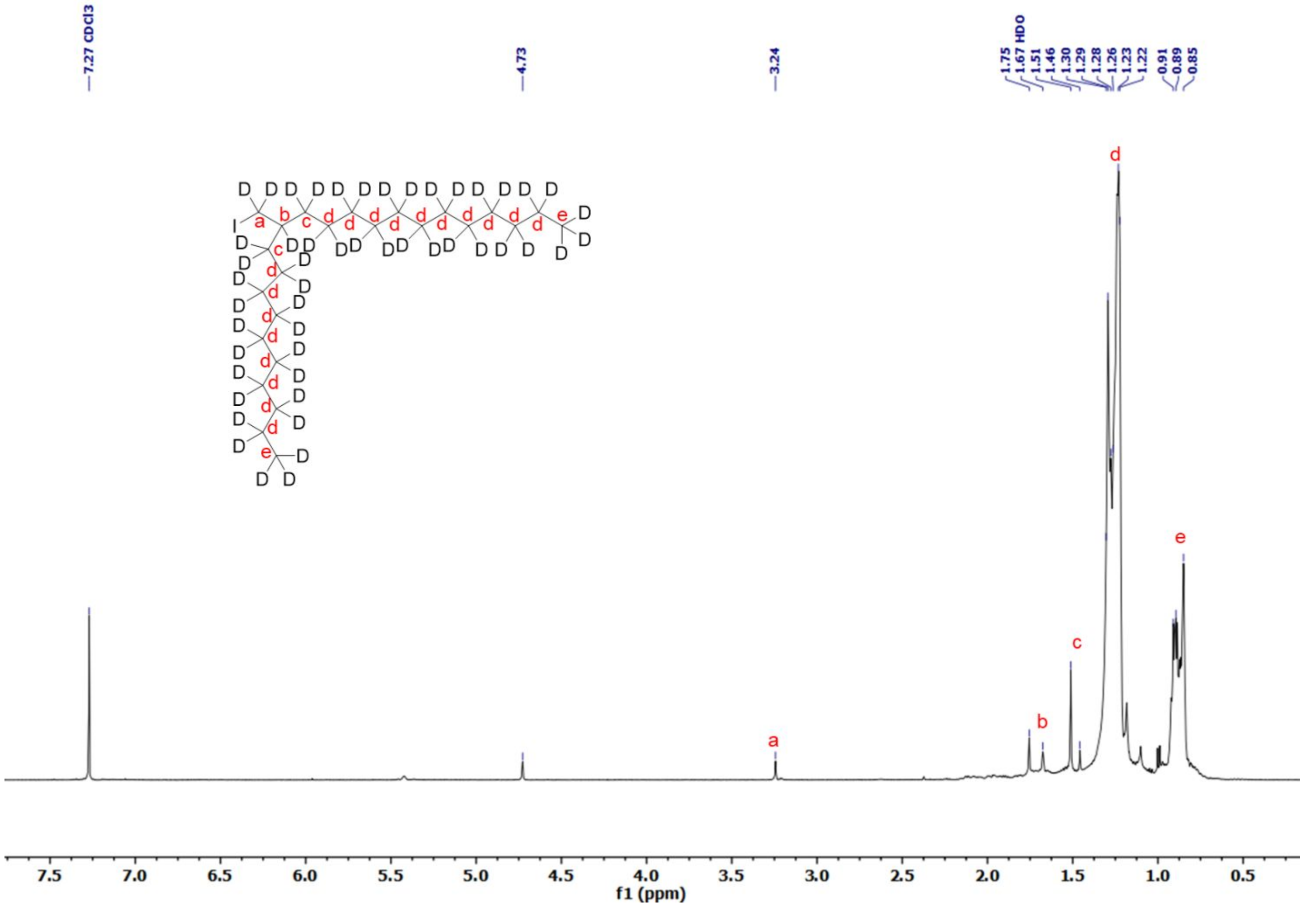

^{1}H NMR spectra of Compound **2c**

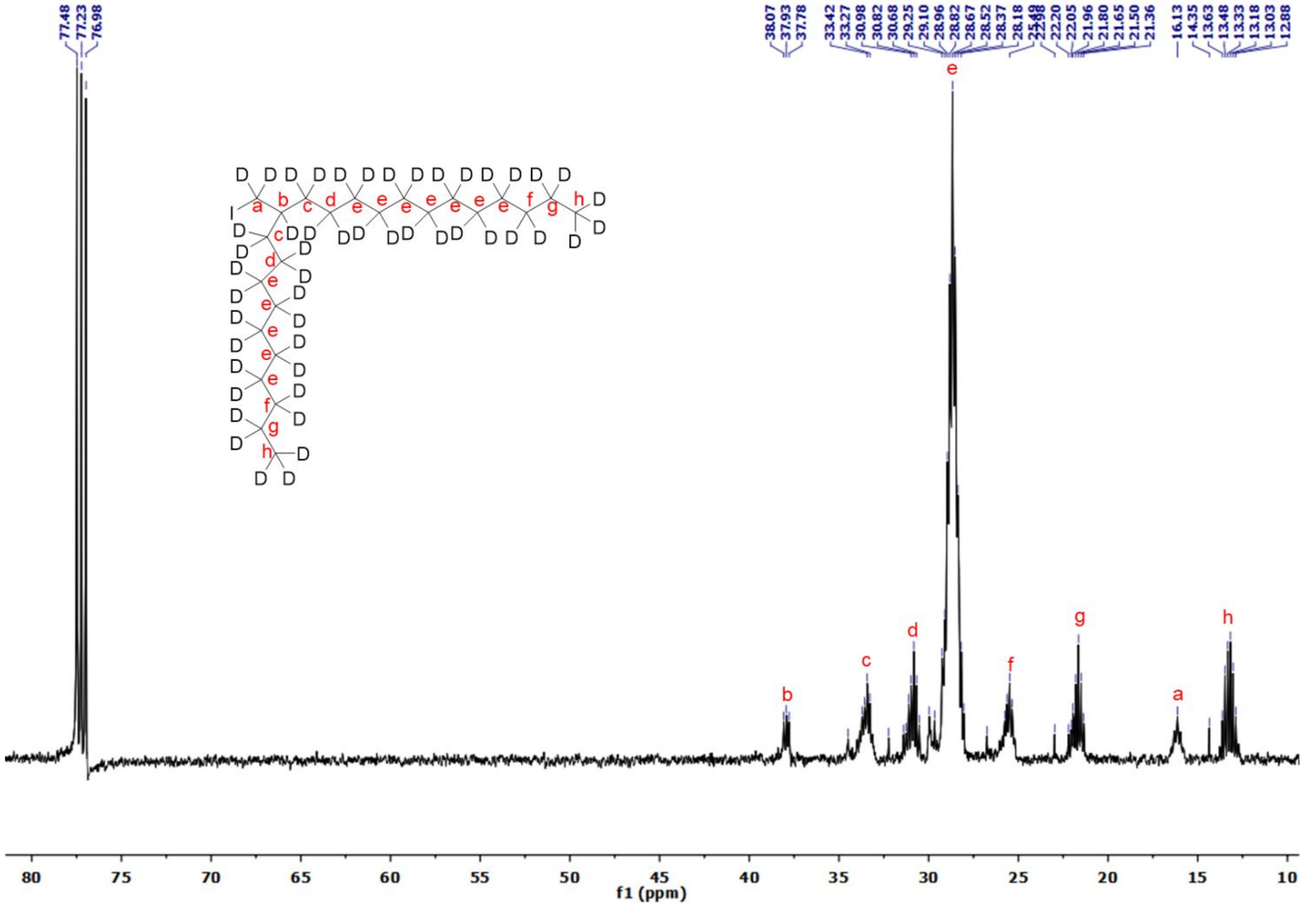

^{13}C NMR spectra of Compound **2c**

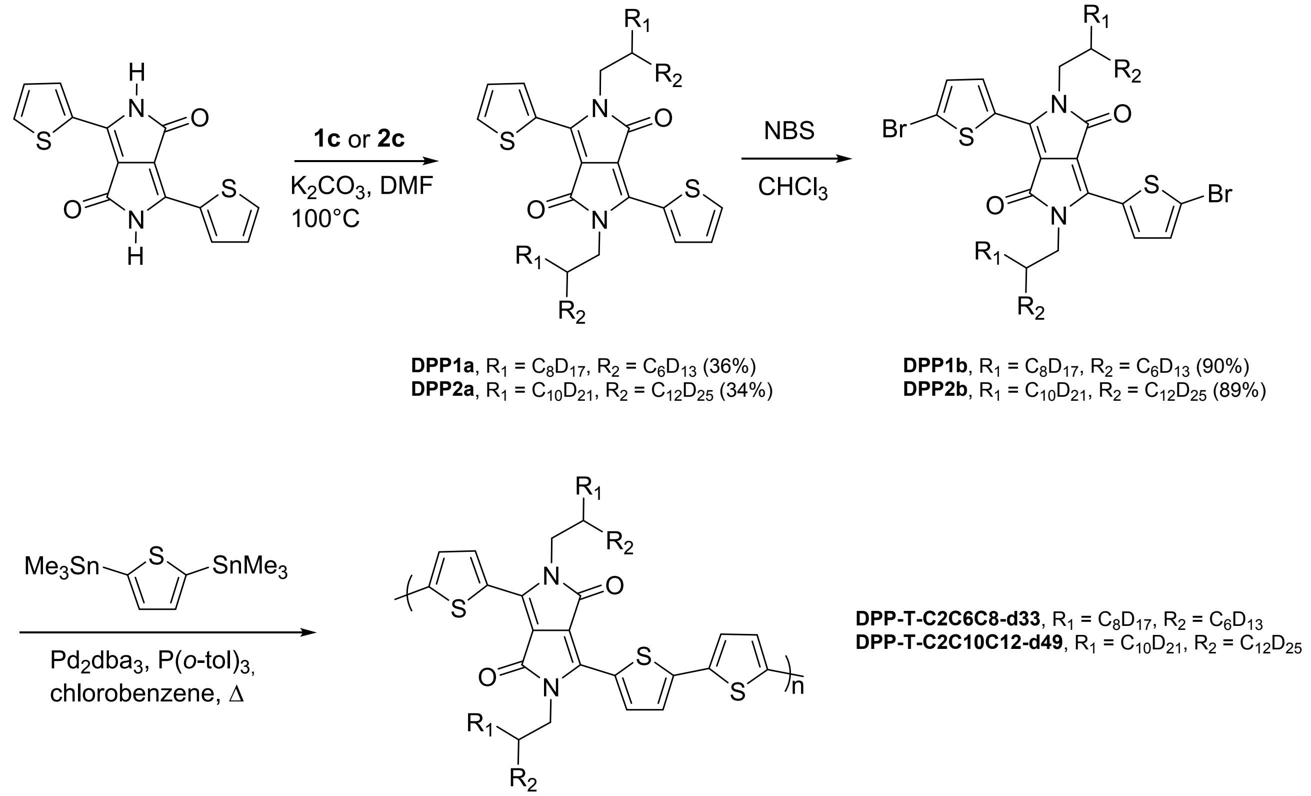

Scheme S2. Synthetic route to deuterated polymers **DPP-T-C2C6C8-d33** and **DPP-T-C2C10C12-d49**

General procedure for DPP alkylation. The synthesis of the DPP-monomers was carried out according to previously reported methods.[1] A round-bottom flask equipped with a magnetic stir bar was purged and charged with 3,6-di(thiophen-2-yl)-2,5-dihydropyrrolo[3,4-c]pyrrole-1,4-dione (DPP), anhydrous potassium carbonate and anhydrous dimethylformamide. The mixture was heated to 100 °C and stirred for 30 minutes under inert atmosphere, then selected alkyl halide was added. The reaction was stirred for 15 hours at 100 °C under inert atmosphere, diluted in chloroform, extracted with H_2O and the organic layer was washed with brine. The organic layer was then dried with Na_2SO_4 and the solvent was removed under reduced pressure. The crude mixture was purified by flash chromatography on silica gel using chloroform as eluent, concentrated under reduced pressure, and precipitated in methanol.

Compound DPP1a. DPP (2 g, 3.6 mmol), DMF (67 mL), K_2CO_3 (2.8 g, 20.0 mmol), **1c** (8.5 g, 16.6 mmol). Yield of **DPP1a**: 34 %. ^{1}H NMR (500 MHz, CDCl3) δ 8.87 (dd, J = 3.9, 1.2 Hz, 2H), 7.61 (dd, J = 5.1, 1.2 Hz, 2H), 7.26 (d, J = 8.8 Hz, 2H), 3.98 (s, 0.02H, residual H in methylene groups), 1.86 (d, J = 6.2 Hz, 0.07H, residual H), 1.27 –1.11 (m, 3H, residual H in methylene groups), 0.78 (d, J = 9.9 Hz, 0.57H, residual H in methyl groups). ^{13}C NMR (500 MHz, $CDCl_3$) δ 161.9, 140.5, 135.3, 130.6, 130.0, 128.5, 108.1, 45.6, 36.8, 30.5, 30.5, 28.6, 28.6, 25.0, 21.9, 13.2. MS: *m/z* calculated for **DPP1a** $[M+H]^+$ 814.9178; found, 814.9135 (16.2%, **DPP1a**-d66), 813.9104 (16.8%, **DPP1a**-d65), 812.9058 (19.7%, **DPP1a**-d64), 811.8990 (18.1%, **DPP1a**-d63), 810.8924 (14.1%, **DPP1a**-d62), 809.8857 (9.4%, **DPP1a**-d61), 808.8790 (5.5%, **DPP1a**-d60).

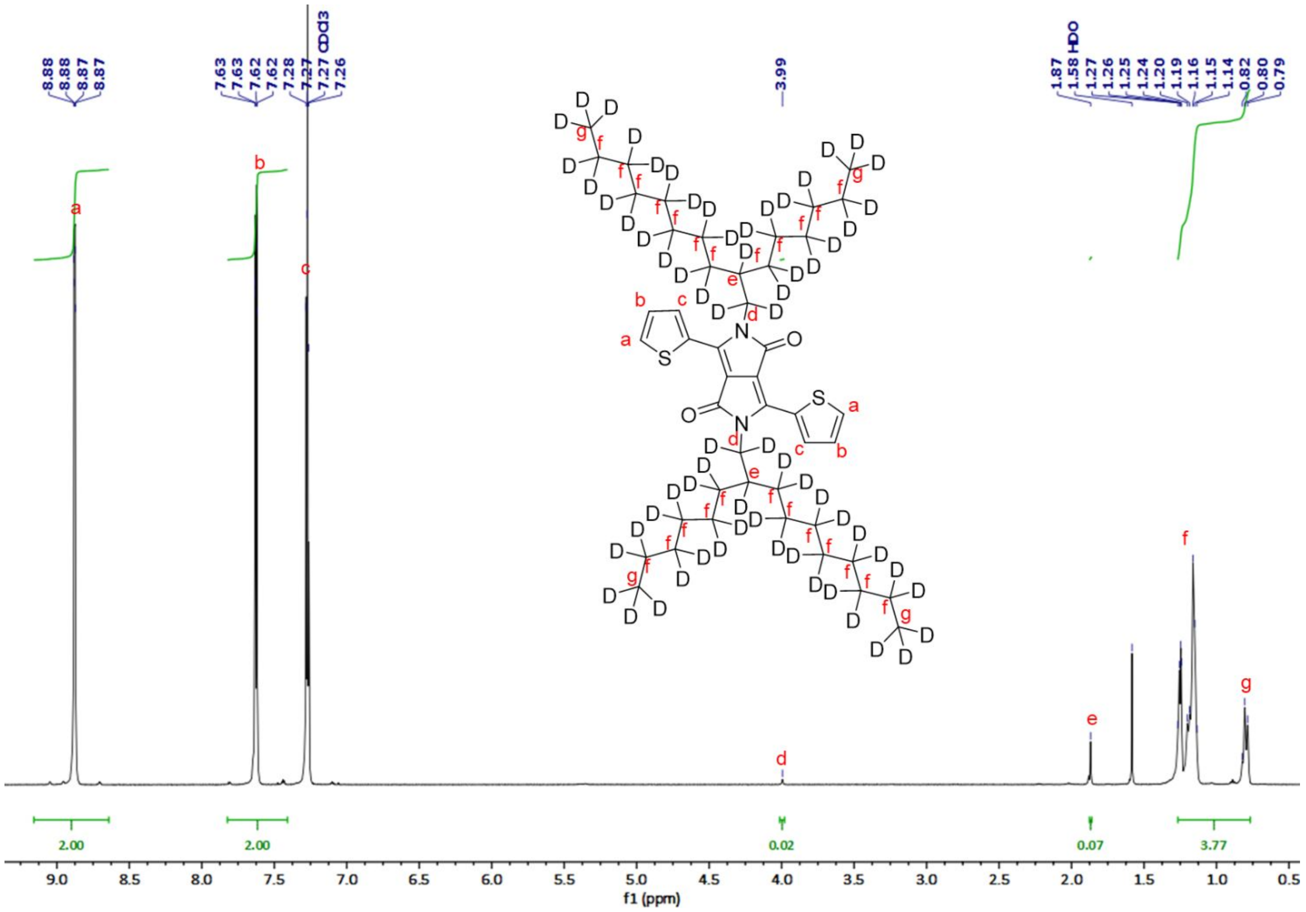

^{1}H NMR spectra of Compound **DPP1a**

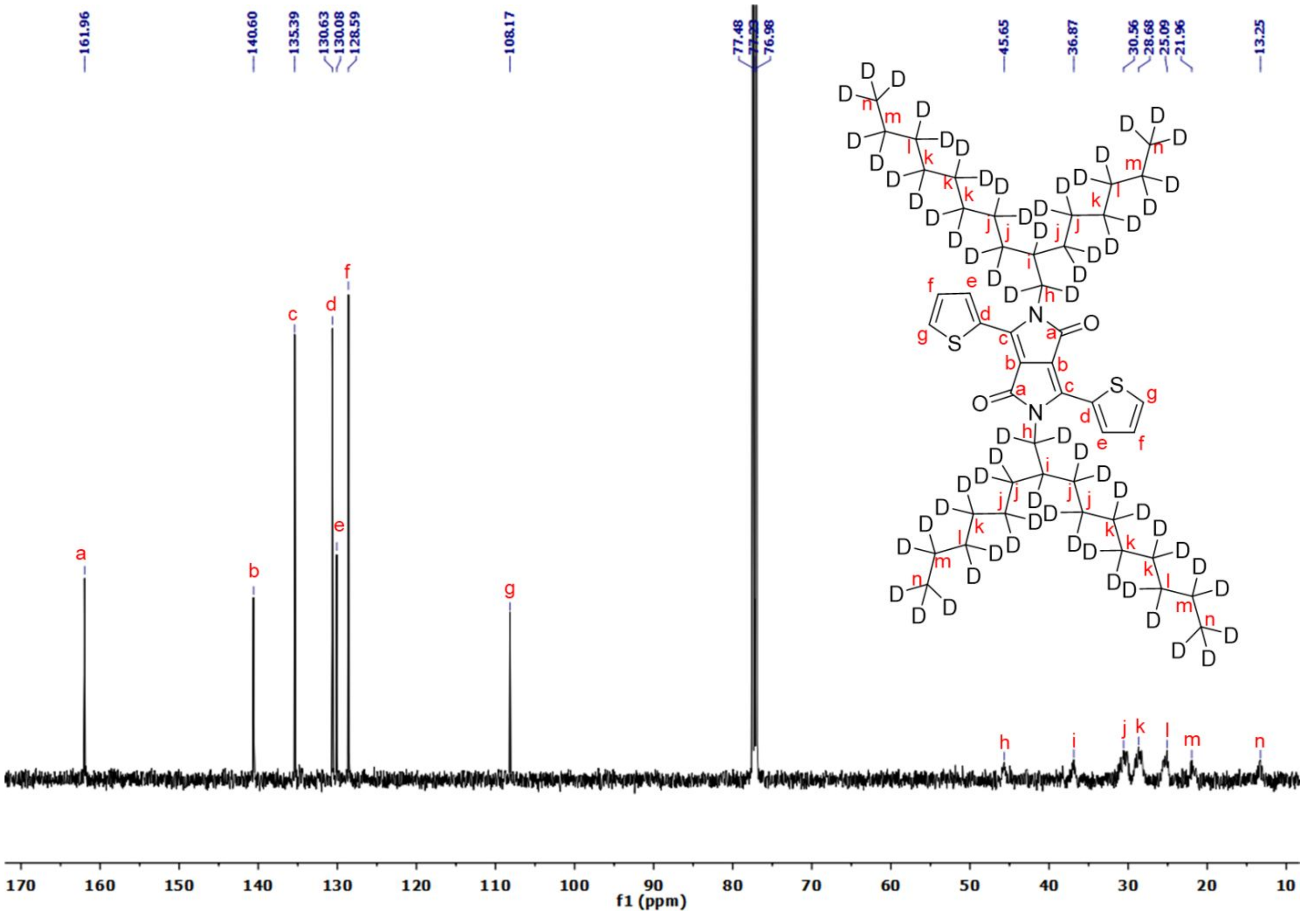

^{13}C NMR spectra of Compound **DPP1a**

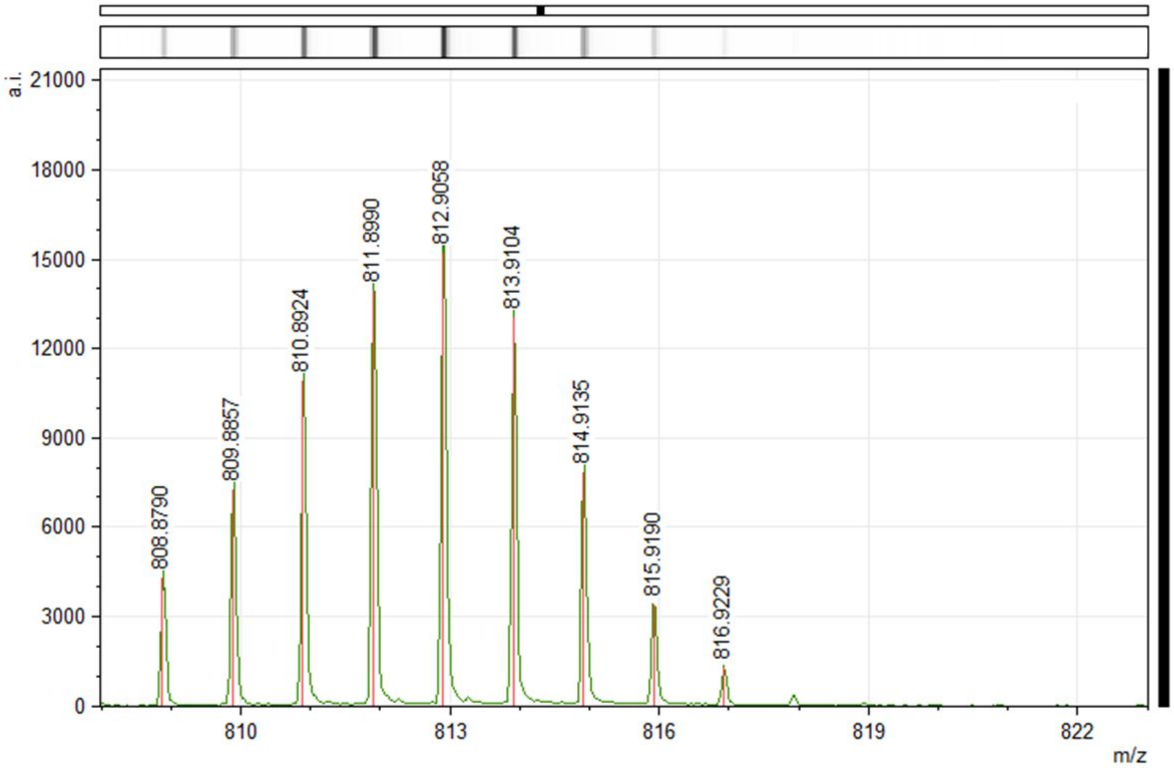

MS spectra of Compound **DPP1a (deuteration level ~96%).**

Compound DPP2a. DPP (2 g, 3.6 mmol), DMF (67 mL), K_2CO_3 (2.8 g, 20.0 mmol), **1c** (6.4 g, 16.6 mmol). Yield of **DPP1a**: 36 %. ^{1}H (500 MHz, CDCl3) δ 8.88 (dd, J = 3.8, 1.2 Hz, 2H), 7.61 (dd, J = 5.0, 1.3 Hz, 2H), 7.30 – 7.23 (m, 2H), 3.98 (s, 0.05H, residual H in methylene groups), 1.86 (d, J = 6.2 Hz, 0.06H, residual H), 1.25 – 1.15 (m, 2.64H, residual H in methylene groups), 0.82 (s, 0.37H, residual H in methyl groups). ^{13}C NMR (500 MHz, $CDCl_3$) δ 161.9, 140.5, 135.3, 130.5, 130.0, 128.5, 108.1, 45.6, 36.9, 30.7, 28.8, 28.7, 28.5, 28.4, 28.2, 25.1, 21.5, 13.4, 13.2, 13.1, 12.9. MS: *m*/*z* calculated for **DPP2a** $[M+H]^+$ 1071.369; found, 1071.3698 (50.2%, **DPP2a**-d98), 1070.3648 (16.3%, **DPP2a**-d97), 1069.3579 (10.8%, **DPP2a**-d96), 1068.3524 (7.2%, **DPP2a**-d95), 1067.3442 (4.8%, **DPP2a**-d94), 1066.3359 (3.3%, **DPP2a**-d93), 1065.3315 (2.7%, **DPP2a**-d91), 1064.3224 (1.8%, **DPP2a**-d90), 1063.3180 (1.6%, **DPP2a**-d89), 1062.3150 (1.2%, **DPP2a**-d88).

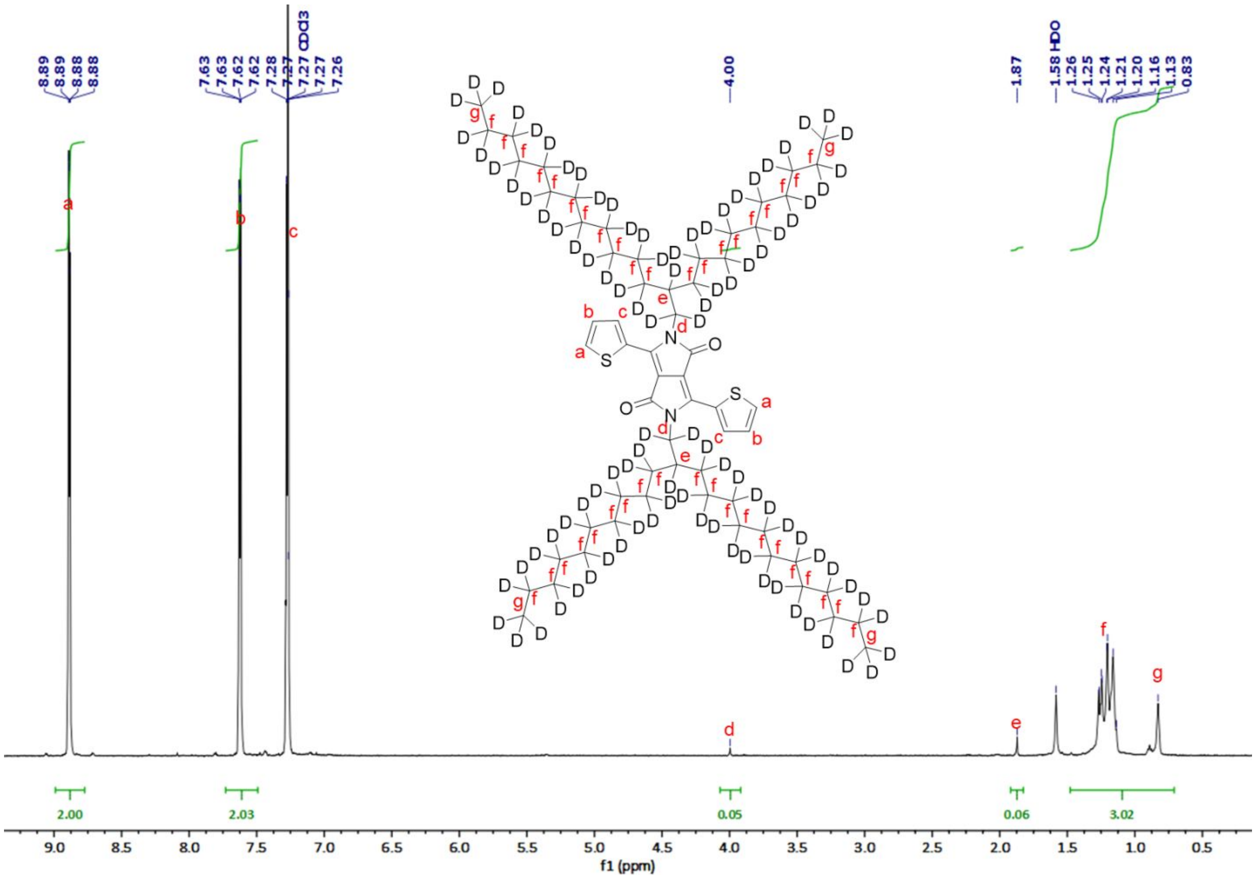

^{1}H NMR spectra of Compound **DPP2a**

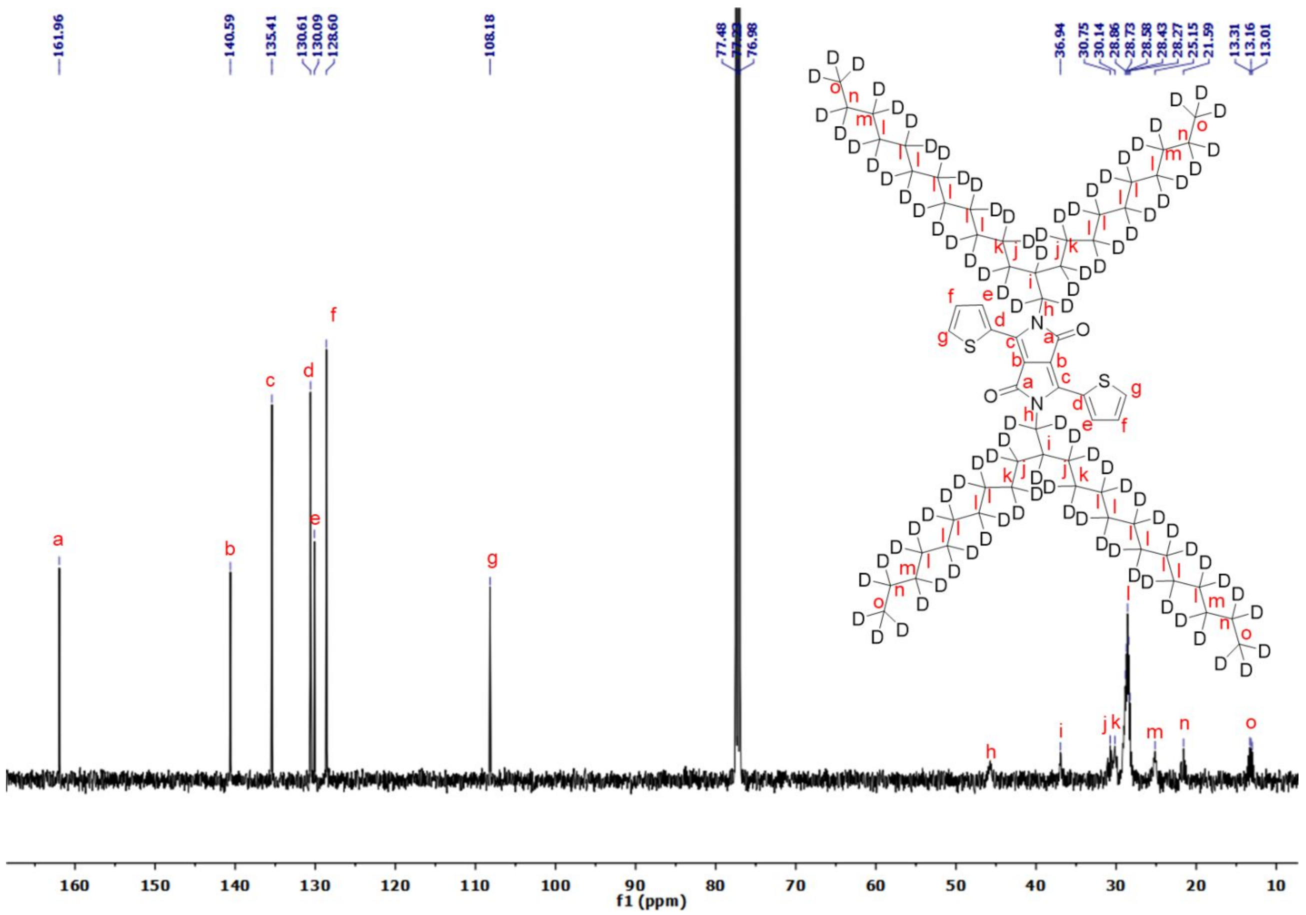

^{13}C NMR spectra of Compound **DPP2a**

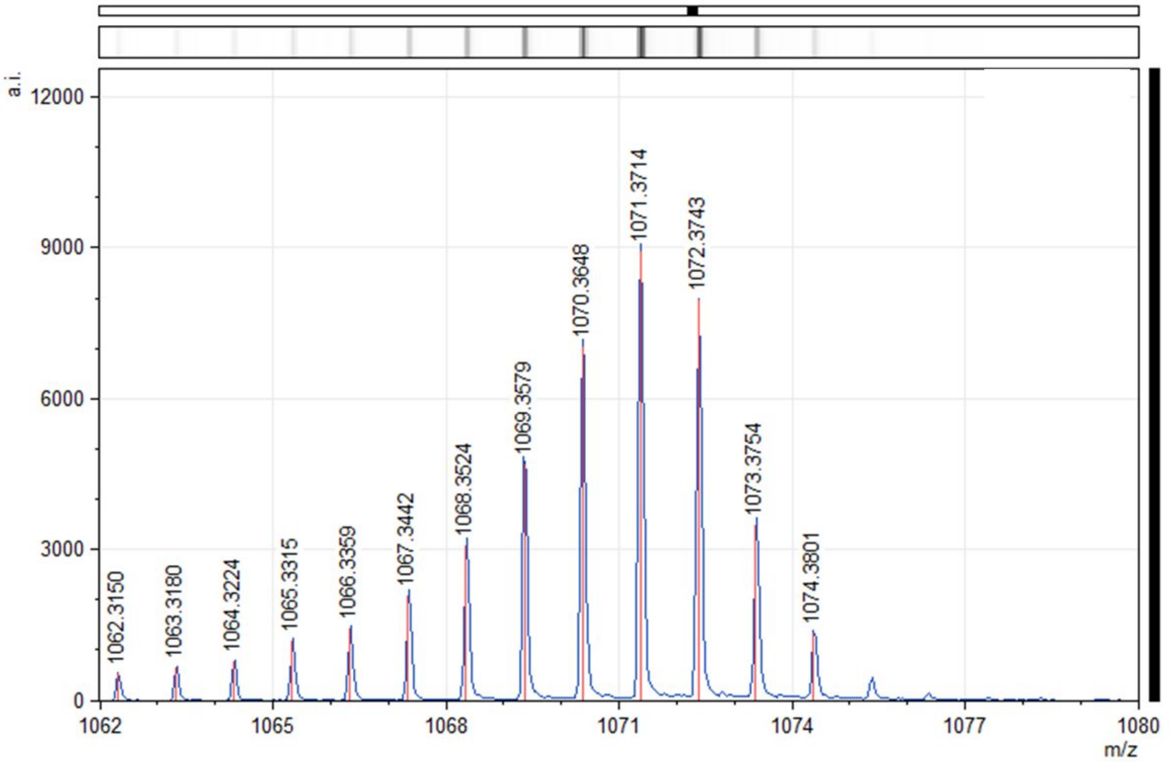

MS spectra of Compound **DPP2a (deuteration level ~98%).**

General procedure for bromination of alkylated DPP. A round-bottom flask was purged and equipped with a magnetic stir bar. The flask was charged with selected alkylated DPP and anhydrous chloroform. *N*-Bromosuccinimide was then added, and the reaction mixture was heated to 60 °C and stirred under inert nitrogen atmosphere. The reaction was monitored by TLC and terminated once no more mono-brominated species were observed. Upon completion, the reaction was extracted with H_2O and dried with Na_2SO_4. The product was then concentrated and precipitated in methanol.

Compound DPP1b. DPP1a (4.0 g, 5.7 mmol), $CHCl_3$ (37 mL), NBS (1.5 g, 8.2 mmol). Yield of **DPP1b**: 89 %. ^{1}H NMR (500 MHz, CDCl3) δ 8.63 (dd, J = 7.2, 4.2 Hz, 2H), 7.29 – 7.18 (m, 2H), 3.89 (s, 0.01H, residual H in methylene groups), 1.83 (s, 0.09H, residual H), 1.28 – 1.16 (m, 2.17H, residual H in methylene groups), 0.80 (s, 0.52H, residual H in methyl groups). ^{13}C NMR (500 MHz, $CDCl_3$) δ 161.6, 139.5, 135.4, 131.6, 131.3, 119.1, 108.2, 77.4, 46.0, 36.8, 30.7, 30.6, 28.5, 28.5, 25.3, 21.5, 13.4. MS: *m*/*z* calculated for **DPP1b** $[M+H]^+$ 972.7445; found, 972.7285 (16.2%, **DPP1b**-d66), 971.7259 (16.8%, **DPP1b**-d65), 970.7226 (19.7%, **DPP1b**-d64), 969.7193 (18.1%, **DPP1b**-d63), 968.7157 (14.1%, **DPP1b**-d62), 967.7117 (9.4%, **DPP1b**-d61), 966.7063 (5.5%, **DPP1b**-d60).

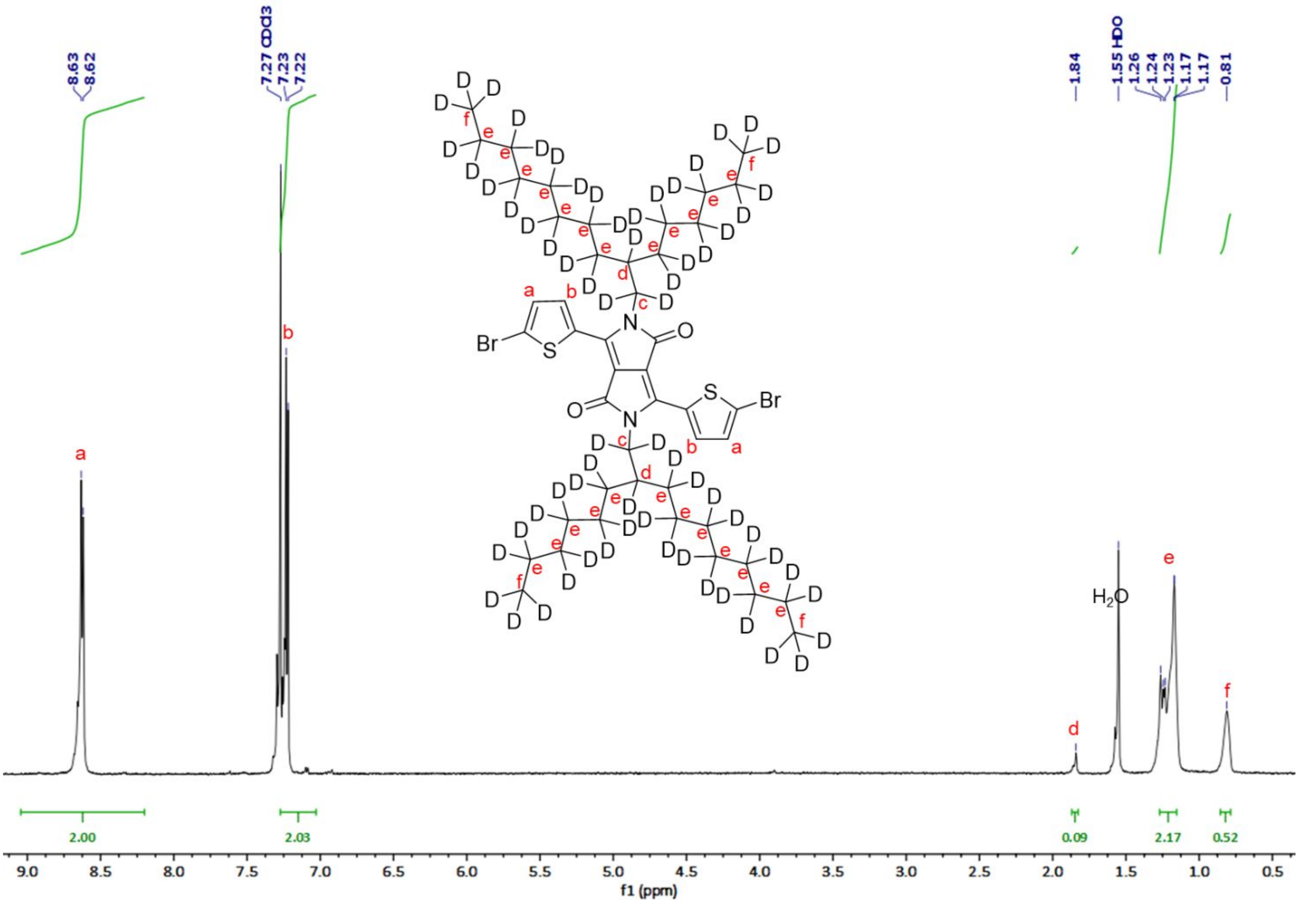

^{1}H NMR spectra of Compound **DPP1b (deuteration level ~ 96%).**

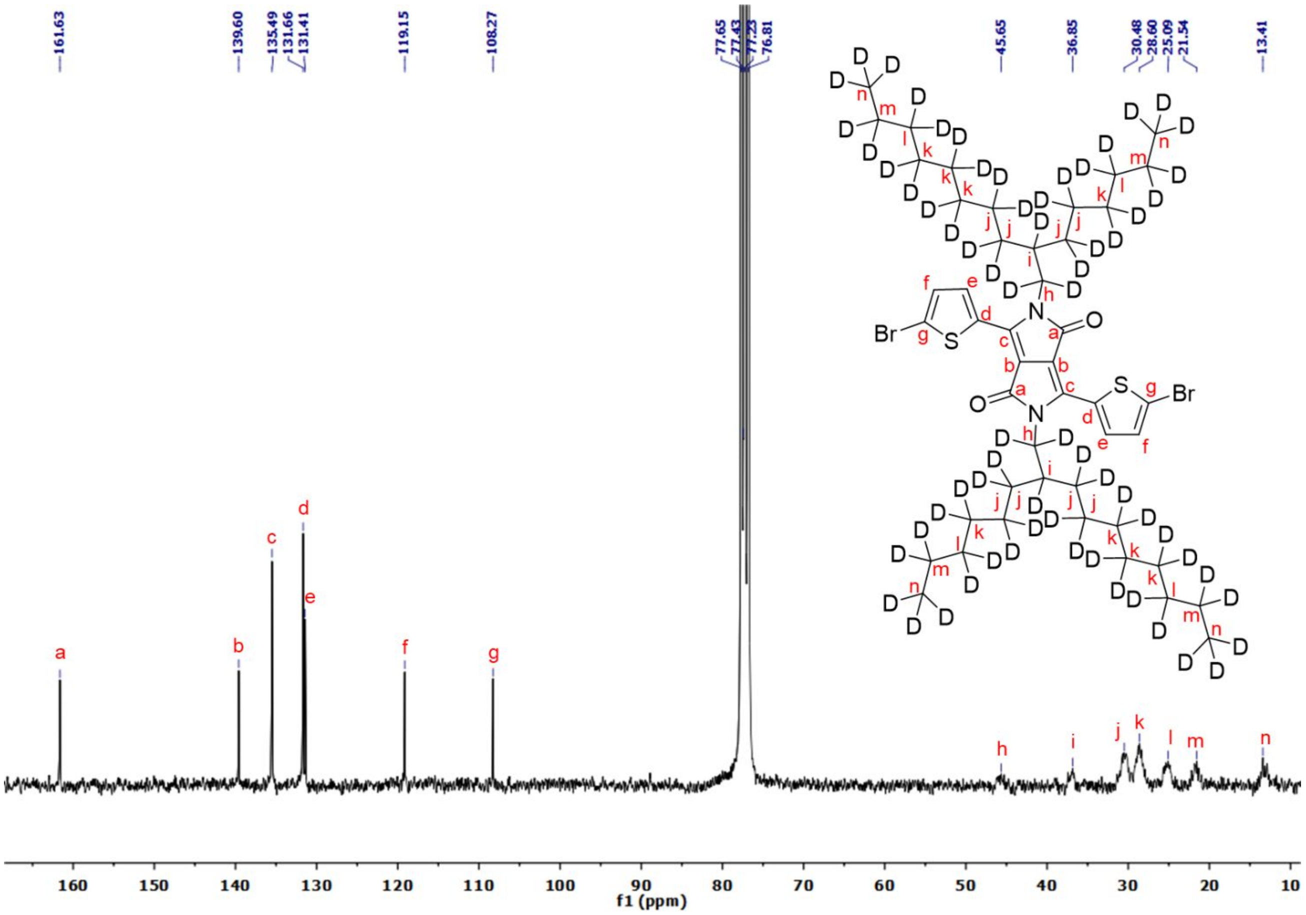

^{13}C NMR spectra of Compound **DPP1b**

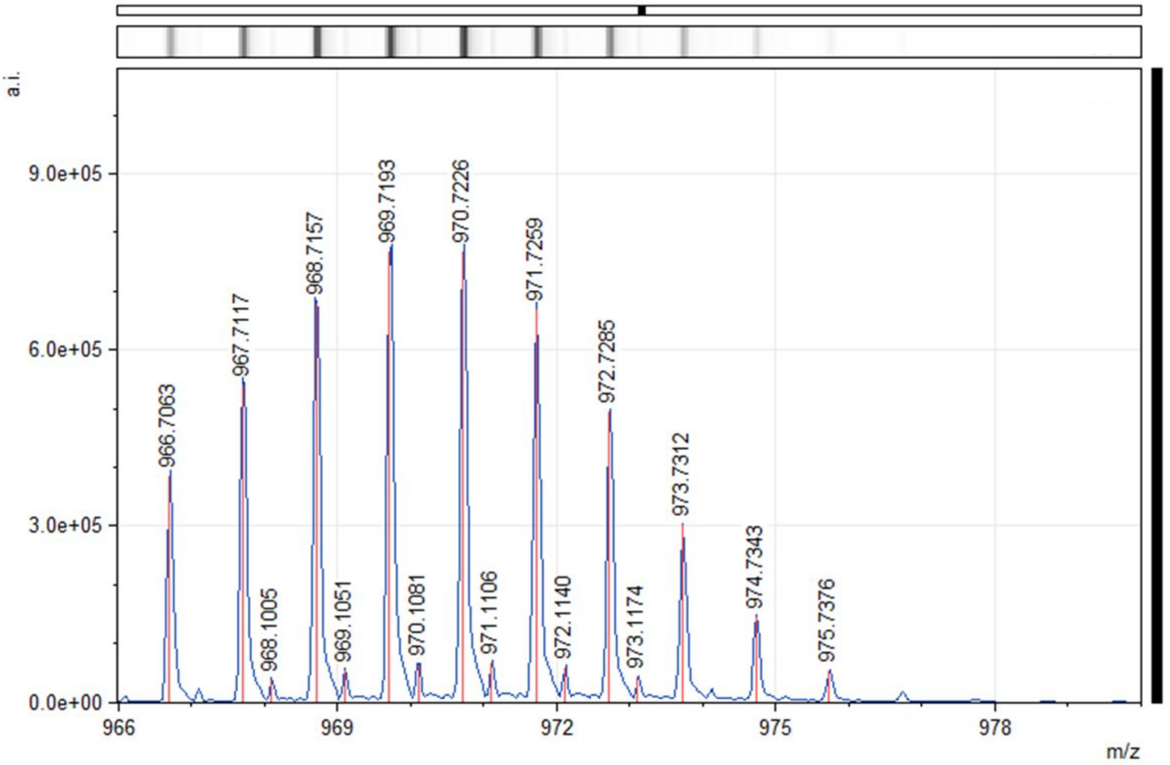

MS spectra of Compound **DPP1b (deuteration level ~ 96%).**

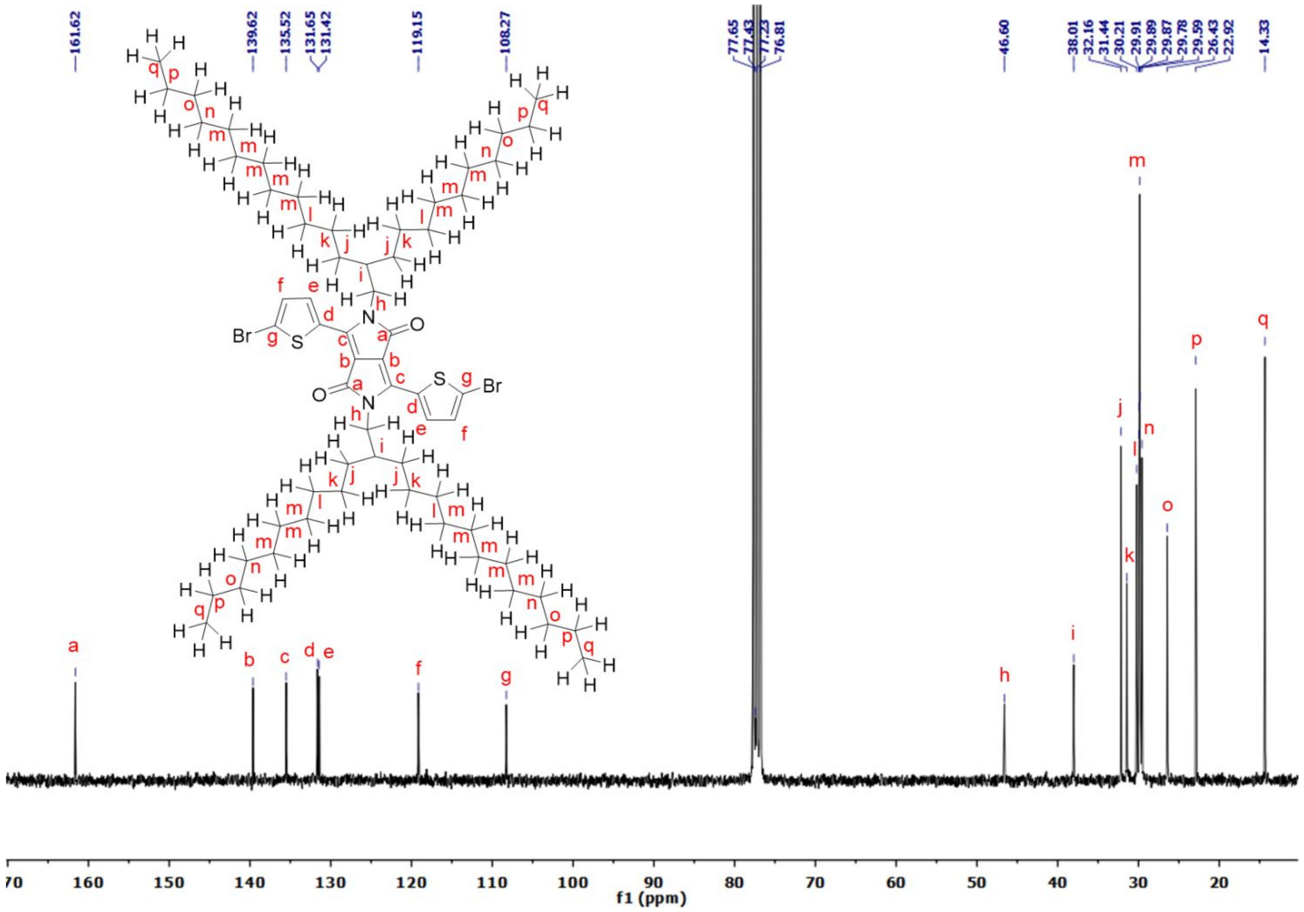

^{13}C NMR spectra of Compound **DPP2b-H**

Compound DPP2b. DPP2a (4.0 g, 7.5 mmol), $CHCl_3$ (37 mL), NBS (2.0 g, 10.7 mmol). Yield of **DPP2b**: 90 %. ^{1}H NMR (500 MHz, $CDCl_3$) δ 8.63 (d, *J* = 4.2 Hz, 2H), 7.22 (d, *J* = 4.2 Hz, 2H), 3.90 (s, 0.01H, residual H in methylene groups), 1.84 (s, 0.08H, residual H), 1.24 – 1.16 (m, 2.22H, residual H in methylene groups), 0.82 (s, 0.24H, residual H in methyl groups). ^{13}C NMR (500 MHz, $CDCl_3$) δ 161.6, 139.5, 135.4, 131.6, 131.4, 119.1, 108.2, 77.6, 77.2, 76.7, 28.5. MS: *m*/*z* calculated for **DPP2b** $[M+H]^+$ 1229.1857; found, 1229.1885 (50.2%, **DPP2b**-d98), 1228.1877 (16.3%, **DPP2b**-d97), 1227.1866 (10.8%, **DPP2b**-d96), 1226.1832 (7.2%, **DPP2b**-d95), 1225.1752 (4.8%, **DPP2b**-d94), 1224.1712 (3.3%, **DPP2b**-d93), 1223.1777 (2.7%, **DPP2b**-d91), 1222.1649 (1.8%, **DPP2b**-d90), 1221.1598 (1.6%, **DPP2b**-d89), 1220.1578 (1.2%, **DPP2b**-d88).

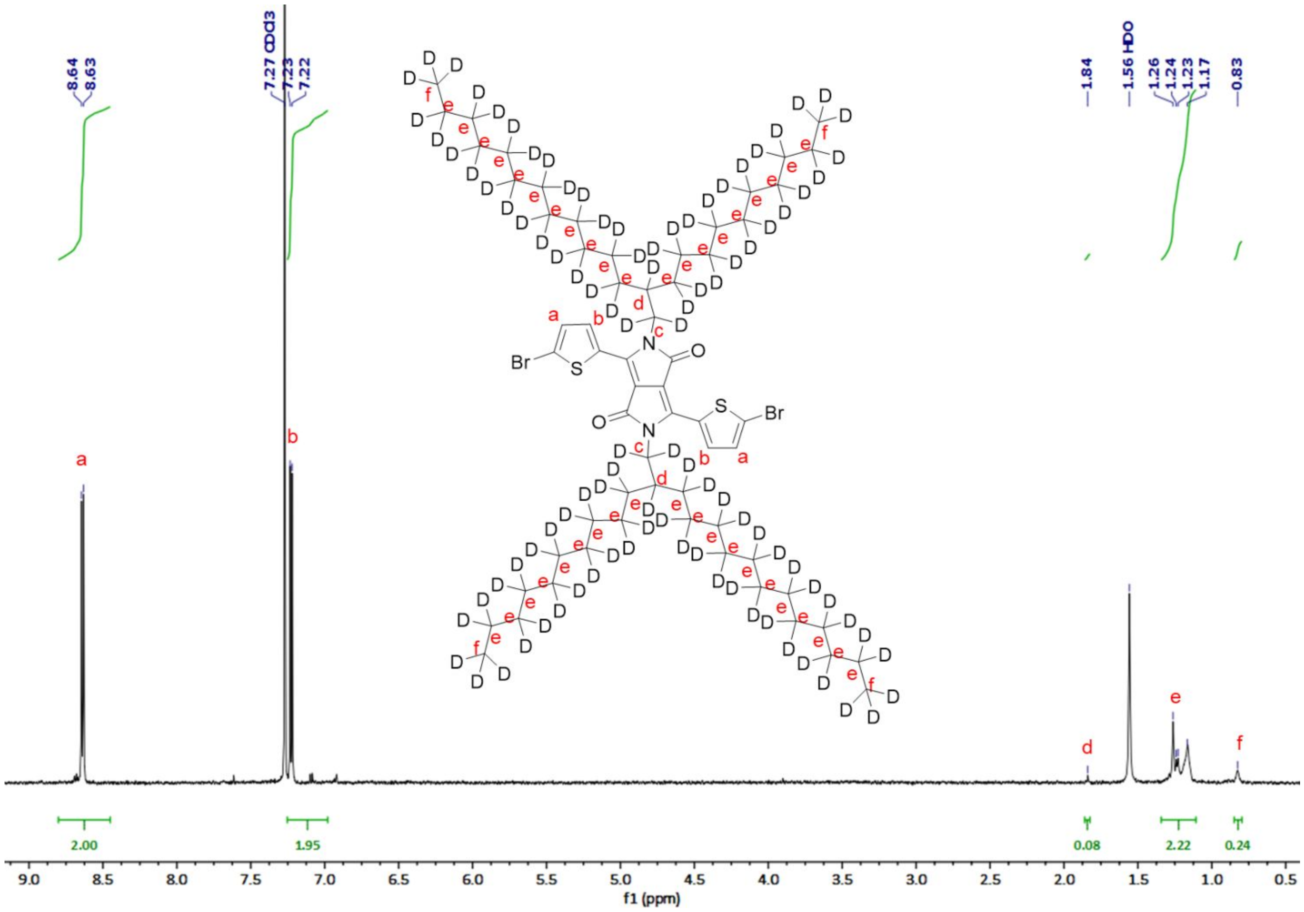

^{1}H NMR spectra of Compound **DPP2b (deuteration level ~ 98%).**

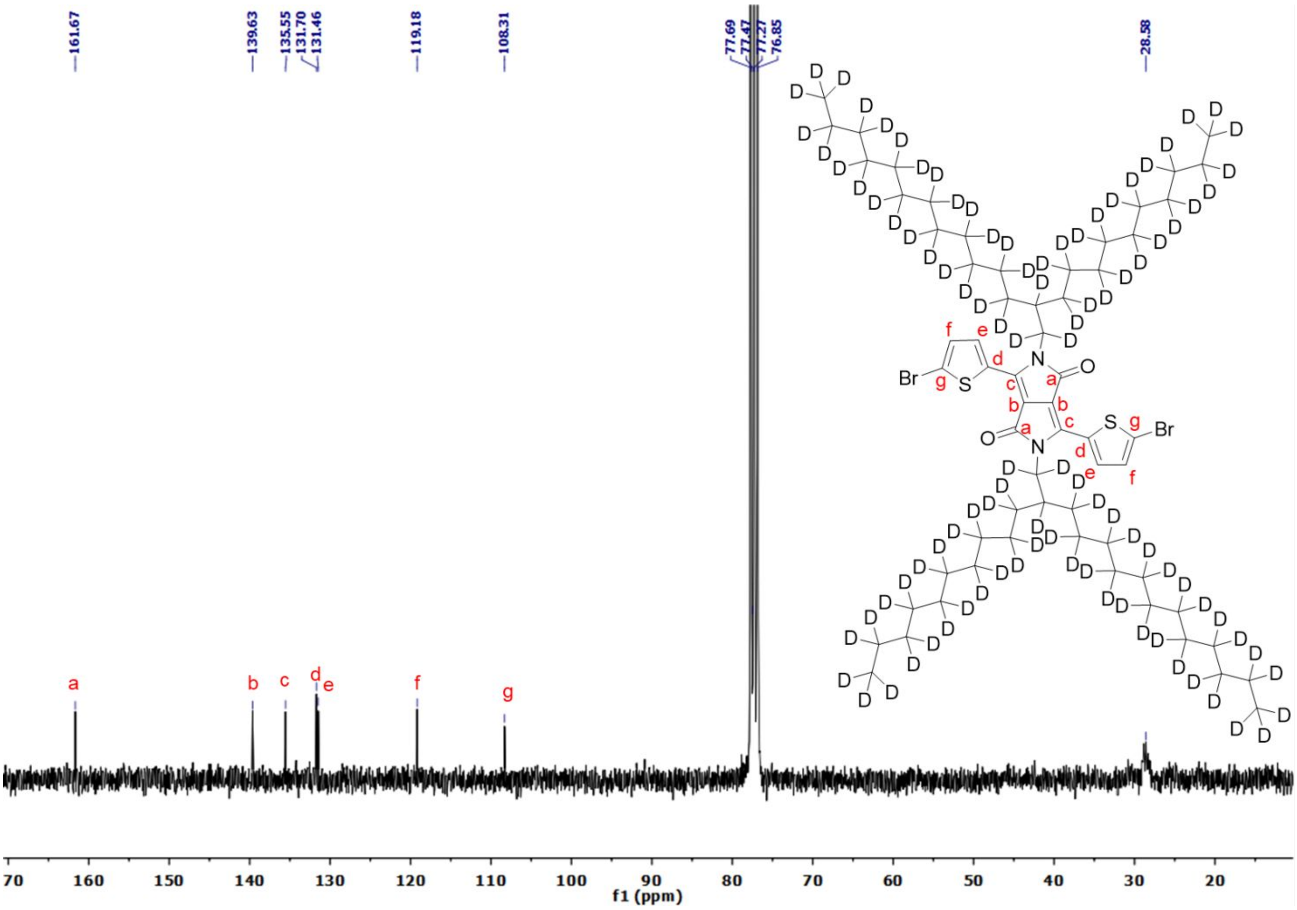

^{13}C NMR spectra of Compound **DPP2b**

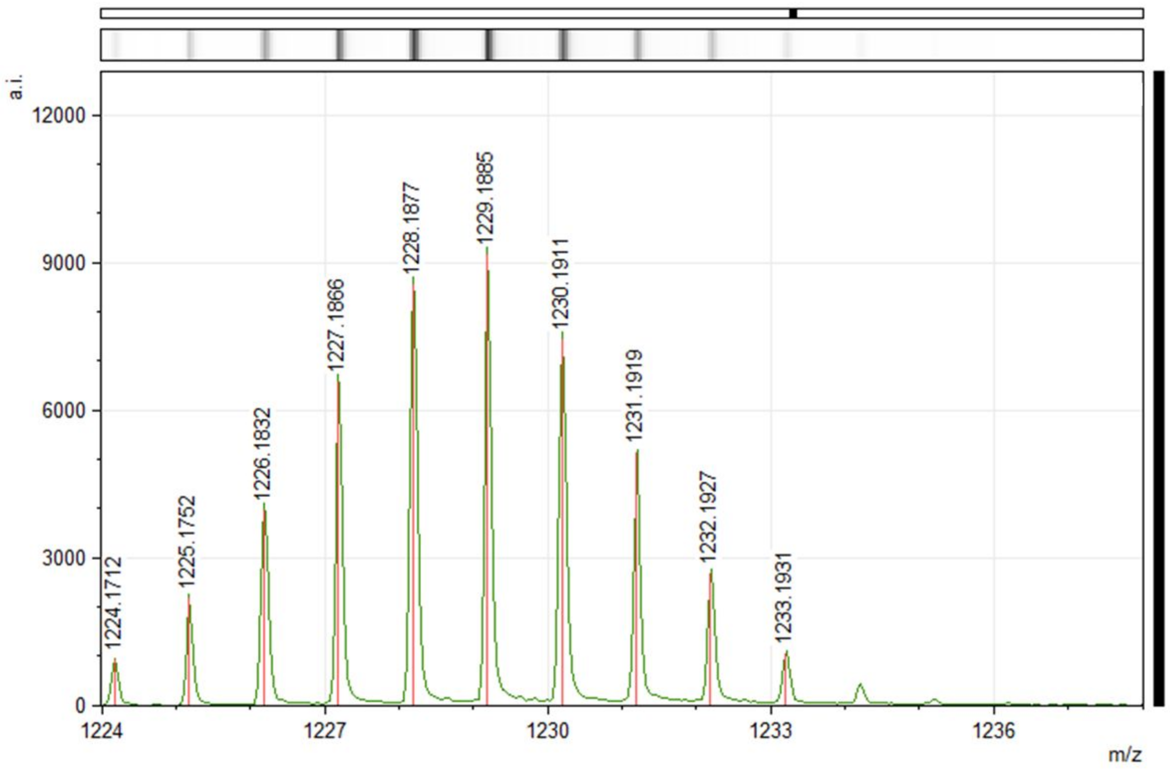

MS spectra of Compound **DPP1b (deuteration level ~ 98%).**

General Procedure for Stille Polycondensation. The synthetic details for DPP-based polymers are described in our previous works.[2] Briefly, a microwave vessel equipped with a stir bar was charged with 2,5-bis(trimethylstannyl) thiophene and selected brominated DPP monomer, followed by the addition of anhydrous chlorobenzene. The solution was then bubbled with N_2 gas for 30 minutes, followed by the addition of catalytic Pd_2dba_3 and P(o-tolyl)$_3$. The vessel was then immediately sealed with a snap cap and microwave irradiated with ramping temperature (Biotage Microwave Reactor; power - 300 W; pressure - 17 bar; stirring – 720; 2 minutes at 100 ^oC, 2 minutes at 120 ^oC, 5 minutes at 140 ^oC, 5 minutes at 160 ^oC, and 40 minutes at 180 ^oC). After completion, the polymer was end-capped with 2-(tributylstannyl) thiophene and 2-bromothiophene successively. The reaction was then cooled to room temperature and precipitated in methanol. The solid was collected by filtration into a glass thimble and the contents were extracted using a Soxhlet apparatus with methanol, hexane, acetone, and chloroform. The high molecular weight fraction was concentrated and precipitated in methanol, followed by filtration and drying under vacuum.

DPP-T-C2C6C8-d33. DPP1b (1.43 g, 1.46 mmol), 2,5-bis(trimethylstannyl) thiophene (0.60 g, 1.46 mmol), chlorobenzene (48.8 mL), Pd_2dba_3 (0.027 g, 0.029 mmol), P(o-tolyl)$_3$ (0.040 g, 0.13 mmol), 2-(tributylstannyl) thiophene (0.55 g, 1.46 mmol), 2-bromothiophene (0.24 g, 1.46 mmol). M_n = 71.7 kDa, M_w = 156.3 kDa, PDI = 2.18

DPP-T-C2C10C12-d49. DPP2b (1.80 g, 1.46 mmol), 2,5-bis(trimethylstannyl) thiophene (0.60 g, 1.46 mmol), chlorobenzene (48.8 mL), Pd_2dba_3 (0.027 g, 0.029 mmol), P(o-tolyl)$_3$ (0.040 g, 0.13 mmol), 2-(tributylstannyl) thiophene (0.55 g, 1.46 mmol), 2-bromothiophene (0.24 g, 1.46 mmol). M_n

= 73.0 kDa, M_w = 210.2 kDa, PDI = 2.88

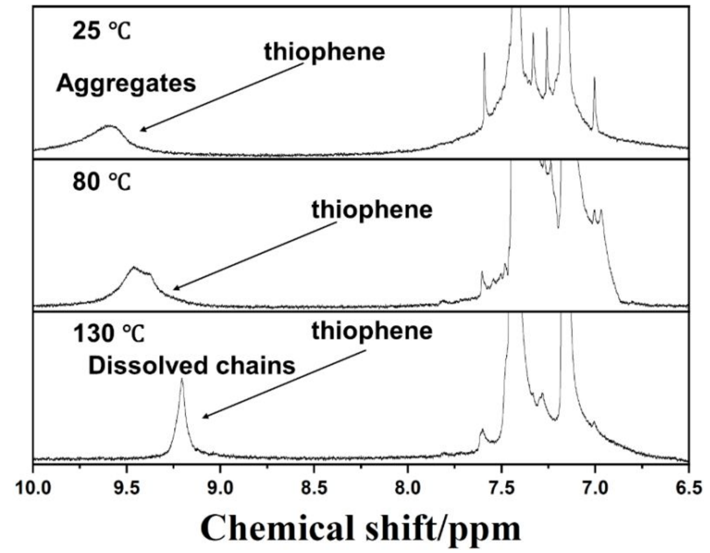

Figure S1. ^{1}H NMR spectra of DPP-T-C2C10C12-d49 in deuterated dichlorobenzene (5 mg ml^{-1}) measured at 130 °C, 80 °C, and room temperature.

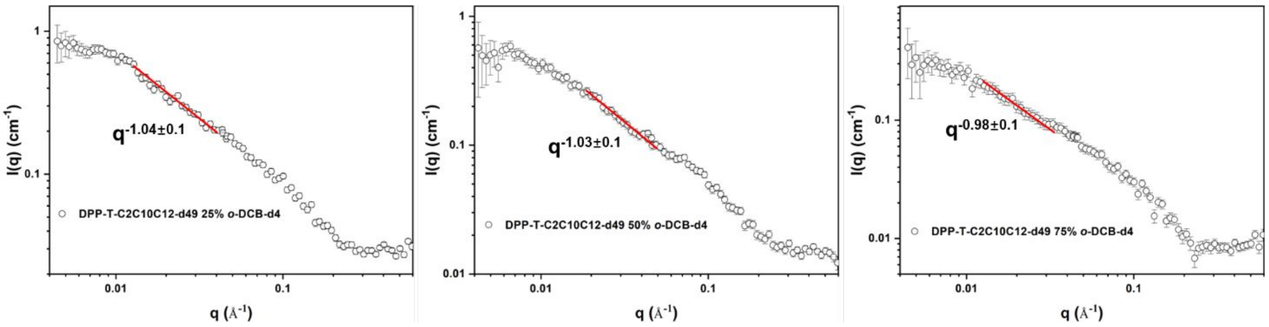

Figure S2. The power law analysis for SANS curves of DPP-T-C2C10C12-d49 at different contrast condition.

Table S1. Parameters obtained from fits to form factors $g_B(q)$ and $g_S(q)$ of DPP-T-C2C10C12-d49 polymers with the flexible cylinder model. Contour length (L_c), persistence length (l_p), and radius (R). And the dispersity for contour length is 1.

Scattering function	L_c (nm)	l_p (nm)	R (nm)
$g_B(q)$	33.8±2.7	18.2±0.1	0.9±0.1
$g_S(q)$	33.8±3.2	18.5±0.1	1.2±0.1

Results for DPP-T-C2C6C8-d33

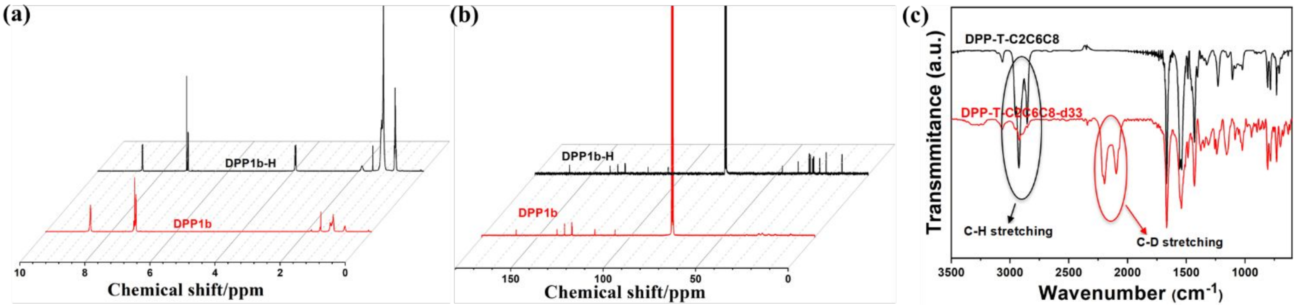

Figure S3. (b) ^{1}H and (c) ^{13}C NMR of DPP-C2C6C8 and DPP-C2C6C8-d33. (d) FTIR spectra of DPP-T-C2C6C8 and DPP-T-C2C6C8-d33.

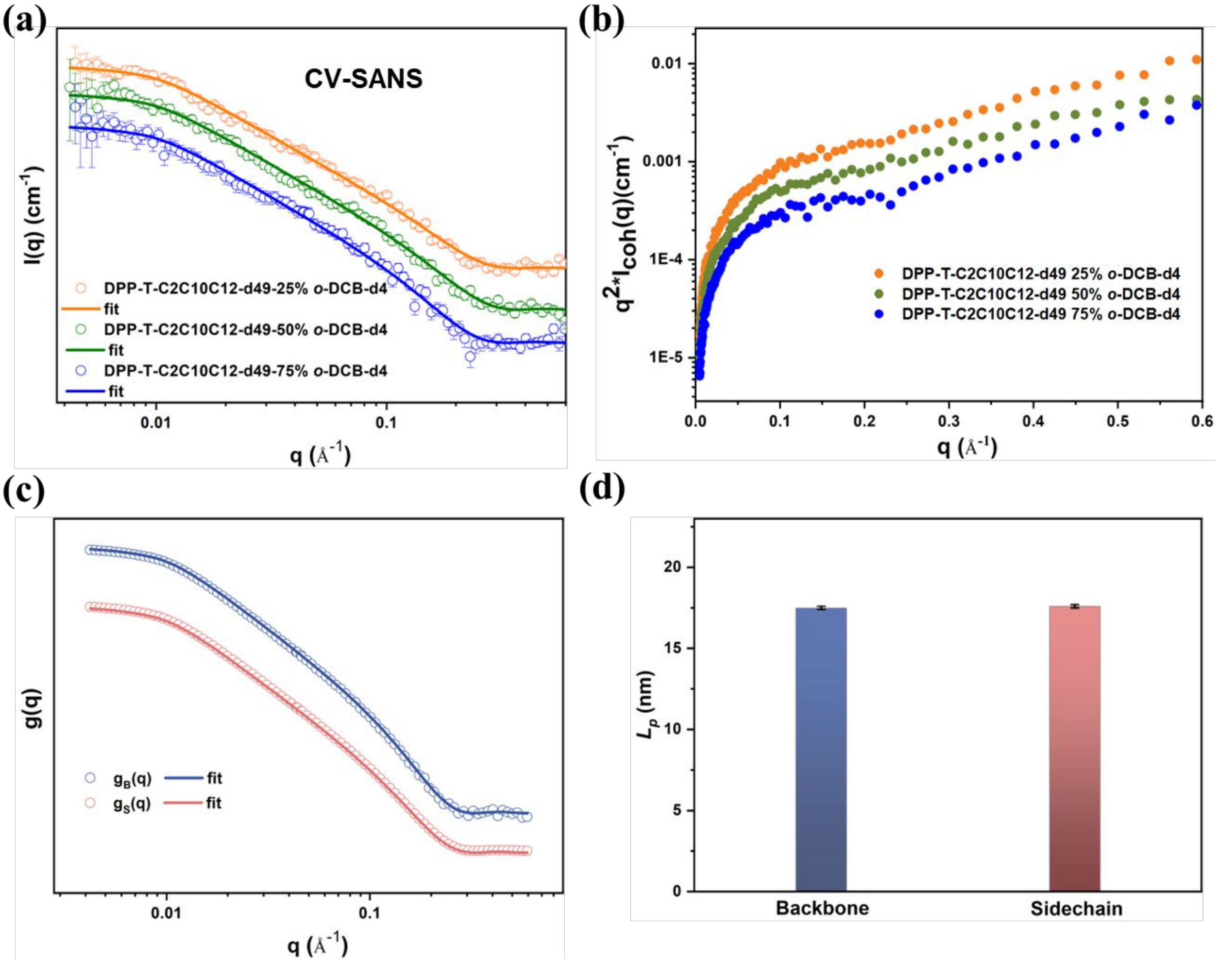

Figure S4. Contrast-variation scattering experiment to probe backbone conformation. (a) Scattering profiles for DPP-T-C2C6C8-d33 from three different CV conditions at a concentration of 5 mg/mL at 130 °C. Solid lines correspond to the best fit to the flexible cylinder model. (c) Kratky plots for DPP-T-C2C6C8-d33 in different CV experiments. (c) Form factors $g_B(q)$ for backbone portion and $g_S(q)$ for side-chain portion. Solid lines correspond to the best fit to the flexible cylinder model. (d) l_p of

backbone, side chain obtained from fitting to correlation functions $g_B(q)$, $g_S(q)$ of DPP-T-C2C6C8-d33 with the flexible cylinder model.

Reduced data was simultaneously fitted to a flexible cylinder model in SasView. SANS Data and the fits using the flexible cylinder model for different contrast conditions are shown in Figure S2 a. The fit results are given in Table S1 below.

Table S2. Parameters obtained from fits to SANS data with the flexible cylinder model. Contour length (L_c), persistence length (l_p), and radius (R) of DPP- T-C2C6C8-d33 polymers. And the dispersity for contour length is 1.

Polymer	L_c (nm)	l_p (nm)	R (nm)
DPP-T-C2C6C8-d33-25% *o*-DCB	31.7±0.1	17.6±3.5	0.9±0.1
DPP-T- C2C6C8-d33-50% *o*-DCB	31.7±0.1	17.7±4.6	0.9±0.1
DPP-T-C2C6C8-d33-75% *o*-DCB	31.7±0.1	17.6±5.0	1.2±0.1

Table S3. Parameters obtained from fits to form factors $g_B(q)$ and $g_S(q)$ of DPP-T-C2C6C8-d33 polymers with the flexible cylinder model. Contour length (L_c), persistence length (l_p), and radius (R). And the dispersity for contour length is 1.

Scattering function	L_c (nm)	l_p (nm)	R (nm)
$g_B(q)$	31.7±1.8	17.5±0.1	1.0±0.1
$g_S(q)$	31.6±1.7	17.6±0.1	1.1±0.1

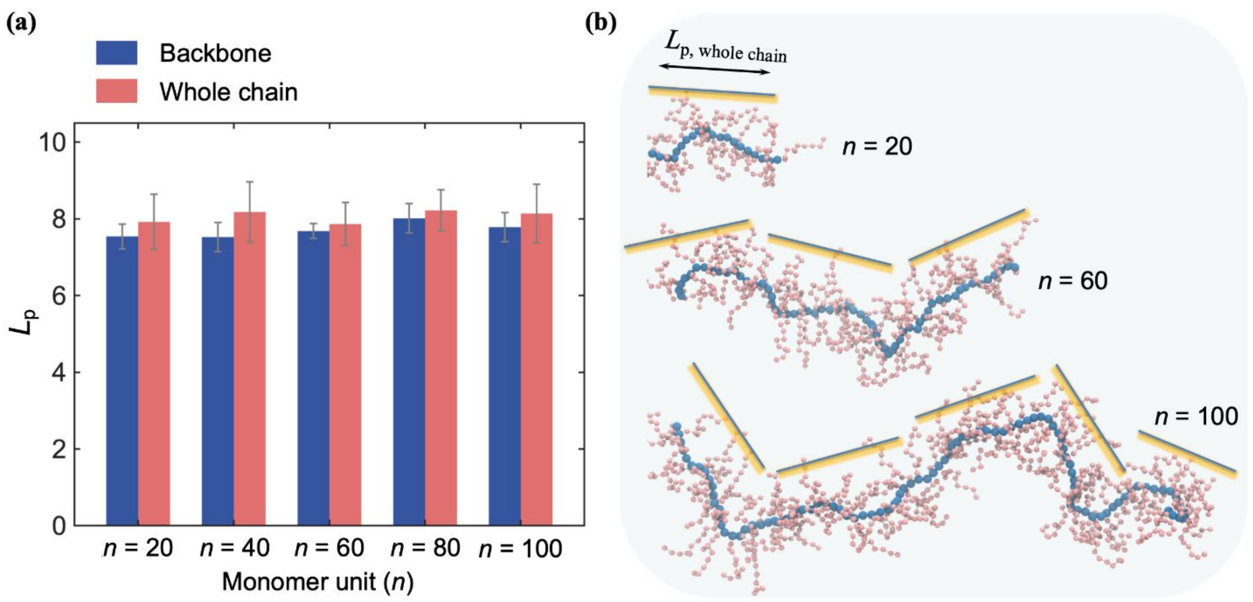

Figure S5. (a) Comparison of the persistence length (L_p) between the backbone alone and the overall polymer chain with increasing number of monomer unit. Backbone stiffness 3.0ε. (b) Representative snapshots of the simulated CP in a solution state with varying chain length.

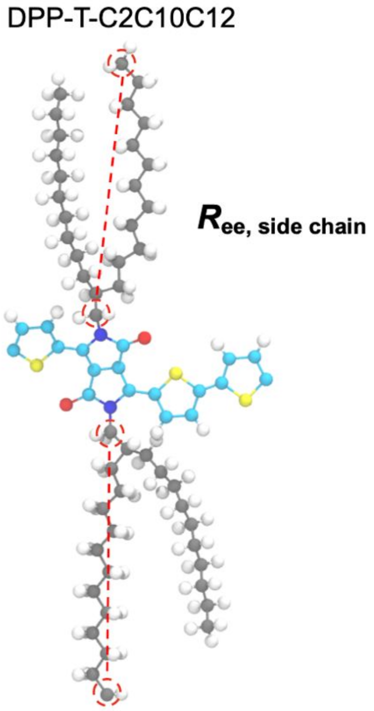

Figure S6~~5~~. Schematic definition of the end-to-end distance of side chain, $R_{ee, side chain}$.

To investigate the conformational behavior of DPP-T-C2C10C12, we analyzed the end-to-end distance of the side chain, $R_{ee, side chain}$, based on the results of our previous all-atomistic MD

simulations.[3] In our study, we observed a $R_{ee,\ side\ chain}$ value of approximately 1.28 nm.

References:

[1] H. Yu, K. H. Park, I. Song, M. J. Kim, Y. H. Kim, J. H. Oh, *J. Mater. Chem. C* **2015**, *3*, 11697–11704.

[2] S. Zhang, A. Alesadi, M. Selivanova, Z. Cao, Z. Qian, S. Luo, L. Galuska, C. Teh, M. U. Ocheje, G. T. Mason, P. B. J. St. Onge, D. Zhou, S. Rondeau-Gagné, W. Xia, X. Gu, *Adv. Funct. Mater.* **2020**, *30*, 1–9.

[3] Z. Cao, Z. Li, S. Tolba, G. T. Mason, M. Xiong, M. U. Ocheje, A. Alesadi, C. Do, K. Hong, T. Lei, S. Rondeau-Gagné, W. Xia, X. Gu, *J. Mater. Chem. A* **2023**, *11*, 12928–12940.
